# Genome sequence of *Anopheles sinensis* provides insight into genetics basis of mosquito competence for malaria parasites

**DOI:** 10.1186/1471-2164-15-42

**Published:** 2014-01-18

**Authors:** Dan Zhou, Donghui Zhang, Guohui Ding, Linna Shi, Qing Hou, Yuting Ye, Yang Xu, Huayun Zhou, Chunrong Xiong, Shengdi Li, Jing Yu, Shanchao Hong, Xinyou Yu, Ping Zou, Chen Chen, Xuelian Chang, Weijie Wang, Yuan Lv, Yan Sun, Lei Ma, Bo Shen, Changliang Zhu

**Affiliations:** 1 Department of Pathogen Biology, Nanjing Medical University, Nanjing, Jiangsu 210029, P.R. China; 2 Jiangsu Province Key Laboratory of Modern Pathogen Biology, Nanjing, Jiangsu 210029, P.R. China; 3 Key Laboratory of Systems Biology, Shanghai Institutes for Biological Science, Shanghai 200031, P.R. China; 4 Office of Research Administration, Nanjing Medical University, Nanjing, Jiangsu 210029, P.R. China; 5 Jiangsu Institute of Parasitic Diseases, Wuxi, Jiangsu 214064, P.R. China

**Keywords:** Genome, Anopheles sinensis, Malaria

## Abstract

**Background:**

*Anopheles sinensis* is an important mosquito vector of *Plasmodium vivax,* which is the most frequent and widely distributed cause of recurring malaria throughout Asia, and particularly in China, Korea, and Japan.

**Results:**

We performed 454 next-generation sequencing and obtained a draft sequence of *A. sinensis* assembled into scaffolds spanning 220.8 million base pairs. Analysis of this genome sequence, we observed expansion and contraction of several immune-related gene families in anopheline relative to culicine mosquito species. These differences suggest that species-specific immune responses to *Plasmodium* invasion underpin the biological differences in susceptibility to *Plasmodium* infection that characterize these two mosquito subfamilies.

**Conclusions:**

The *A. sinensis* genome produced in this study, provides an important resource for analyzing the genetic basis of susceptibility and resistance of mosquitoes to *Plasmodium* parasites research which will ultimately facilitate the design of urgently needed interventions against this debilitating mosquito-borne disease.

## Background

Malaria is caused by infection with *Plasmodium* parasites, which are transmitted via the bites of infected female *Anopheles* mosquitoes [1]. Malaria is prevalent and widely distributed in tropical and subtropical regions, including much of sub-Saharan Africa, Asia, and the Americas [2,3]. Indeed, according to the latest World Malaria Report, in 2010 malaria caused an estimated 216 million clinical episodes and 655,000 deaths worldwide [4]. Of the few available management strategies for this disease, vector control offers an important means of limiting the spread of malaria. The effective control of mosquito vectors, however, requires information on their genetic structure, because the biology and physiology of infections, the development of insecticide resistance, and the epidemiology of malaria in the human host can all be affected by genetic variation in the mosquito vector populations. To date, our understanding of the role of vector genetics in the dynamics of malaria transmission is poor. In particular, the function and evolutionary aspects of important genes, such as those associated with vector competence, remains unclear. The paucity of genetic information on *Plasmodium*-susceptible mosquitoes is a major obstacle to the development of appropriate diagnostic and therapeutic tools against malaria.

All malaria vectors belong to the subfamily *Anophelinae*. Mosquitoes of the subfamily *Culicinae* are not susceptible to infection by *Plasmodium* parasites and thus, do not transmit *Plasmodium*. The genomes of *A. gambiae*, *Aedes aegypti* and *Culex quinquefasciatus* were sequenced in 2002, 2007 and 2010, respectively. Comparative genomic studies of these three species have provided important genetic insights into this vector-disease system including the identification of conserved gene regions; the identification of highly diverged genes; recognition of gene families that have expanded or contracted; and the evolution of species-specific physiological or behavioral genetic variations. Nevertheless, information provided by these genome sequences has provided only a limited understanding of the genetic basis of species-specific susceptibility to *Plasmodium*.

In this study, we sequenced the genome of *A. sinensis*, a malaria vector within the subfamily *Anophelinae. A. sinensis* is an Asiatic mosquito species with a wide geographical distribution in East Asia region, ranging from the Philippines to Japan [5]. While *A. gambiae* is considered to be an efficient vector of *P. falciparum*[6], *A. sinensis* is suspected to be the most dominant and important vector of *P. vivax*[7]. In addition, *A. sinensis* was found to be solely responsible for the recent outbreaks of malaria in China [8]. Contrasting the genetic composition of these two anopheline mosquitos with that of culicine mosquitos offers a means of investigating the genetic basis of their phenotypic differences to *Plasmodium* susceptibility, which is a critical step in developing novel ways to reduce human malaria transmission.

Traditional methods of gene detection are costly and time consuming and typically require prior knowledge of target gene regions, as they rely on specific primers. Therefore, these techniques are unsuitable for analyzing large numbers of unknown sequences. The development of next-generation sequencing (NGS) technologies provides an ideal method for rapid and reliable genomic exploration of mosquitoes.

In this study, we employed Roche/454 GS FLX sequencing technology to produce the first genome sequences of *A. sinensis*. A single-end 454 Jr. run combined with a paired-end 454 Jr. run (3, 8 and 20 Kb libraries) provided a cost-effective solution that produced high quality draft assemblies, and allowed us to obtain detailed gene annotations and meaningful results. Our comparative genomic analyses of the genomes of anopheline and culicine mosquitoes revealed key genetic difference that may underlie important species-specific biological functions in these two groups. This study provides critical genomic information that will pave the way for further in-depth molecular investigations into the biological and vector competency of *A. sinensis*.

## Results and discussion

### Sequencing and assembly

We sequenced the whole-genome of *A. sinensis* using the Roche/454 GS FLX sequencing approach. A total of 5,171,177 single-end reads, 6,302,769 3 Kb mate-pair reads, 2,829,232 8 Kb mate-pair reads and 864,365 20 Kb mate-pair reads were generated (Table 1). After adaptor trimming and low quality reads filtering, a total of 2.7 G single-end sequences and 0.6 G mate-pair sequences were obtained. The genome size of *A. sinensis* was estimated 267.7 Mb based on K-mer statistics (Table 2), supporting previous estimates of genome size in this mosquito subfamily (230-284 M) [9].

**Table 1:** **Summary of the raw reads of the sequencing analysis of *A. sinensis***

**Library**	**Raw reads**	**Trimmed reads**	**Reads used in assembly**	**Average read length (bp)**
Single-end	5,171,177	153,380	5,017,797	383
3 K paired end	6,302,769	270,609	6,032,160	205
8 K paired end	2,829,232	80,660	2,748,572	207
20 K paired end	864,365	14,201	850,164	347

**Table 2:** **Estimated genome size of *A. Sinensis *** **based on K-mer analysis**

**K-mer value**	**K-mer number**	**Depth**	**Genome size (bp)**	**Used bases**	**Used reads**
13	1,874,145,919	7	267,735,131	1,934,235,439	5,007,460

The whole-genome assembly initially resulted in 9597 scaffolds. After screening for contamination, three scaffolds were identified as putative contaminating sequence of possible bacterial origin and removed (Additional file 1: Table S1). The final 9594 scaffolds spanned 220.8 M with an N50 scaffold size of 814.2 Kb, and contained approximately 82.5% of the *A. sinensis* genome, based on a genome size of 267.7 Mb. Contig sizes ranged from 65 bp to 357,810 bp, while scaffold sizes ranged from 75 bp to 5,918,260 bp (Table 3). Assembly quality was assessed by aligning the transcripts onto the scaffolds, and 97.5% mapping rate was observed (Additional file 1: Table S2). Assembly quality was also assessed by aligning 454 single reads to the scaffolds. Approximately 99.2% of single 454 data with depth over 3X can be mapped. Further analysis of single nucleotide variants (SNVs) and insertion and deletion (INDEL) variation revealed base error rate was 0.015% and short indel error rate was 0.011%, which supported the high quality of genome assembly (Additional file 1: Table S3). Additionally, analysis of the draft genome assembly for core eukaryotic genes (CEGs) revealed almost all of 458 CEGs (446 out of 458, 97.4%), complete 248 highly conserved CEGs (239 out of 248, 96.4%) and partial 248 highly conserved CEGs (244 out of 248, 98.4%) were found, again confirming the assembly quality of *A. sinensis*. This Whole Genome project has been deposited at DDBJ/EMBL/GenBank under the accession ATLV00000000. The version described in this paper is version ATLV01000000.

**Table 3:** **Statistics for the assembly of the *A. sinensis *** **genome**

	**Contig**		**Scaffold**	
	**Size (bp)**	**Number**	**Size (bp)**	**Number**
N90	2,384	10,962	30,600	582
N80	7,384	6,003	149,975	249
N70	13,407	3,858	338,010	149
N60	20,357	2,558	537,812	98
N50	30,137	1,685	814,231	66
Longest	357,810		5,918,260	
Total Size	214,524,114		220,784,734	
Total Number (>100 bp)		27,488		9,596
Total Number (>2 Kb)		12,156		2,038

This genome had a GC percentage of 42.6%, which was higher than the mean GC content in the other three sequenced mosquito species (Table 4). Earlier study suggested that the amount of introns may have an association with differential GC content among these three sequenced mosquito species [10]. Here, we found relatively lower introns in *A. sinensis* (~32,000 introns) than other three mosquito species (*A. gambiae* ~ 38,000 introns, *A. aegypti* ~ 51,000 introns, and *C. quinquefasciatus* ~ 52,000 introns). This result strongly suggested a negative correlation between the GC content and intron numbers. Haddrill et al. [11] found a strongly negative correlation between intron length and rate of divergence of the genes. Also, a positive correlation between the recombination rate and GC content has been found in many species, such as yeast [12], birds [13], insect [14], plants [15] and mammals [16,17]. Recombination acquire a larger amount of genetic diversity [18]. Both lower intron length (Additional file 1: Table S4) and higher GC content in *An. sinensis* and *An. gambiae* may indicate high genetic diversity rate than other two subfamily Culicinae mosquitoes. Interestingly, genetic diversity in the susceptibility to malaria parasites in mosquitoes has been already amply confirmed [19,20]. However, we also recognized this estimate of GC content was susceptible of a non trivial error bar, because nearly 20% of the genome was missing from the draft assembly.

**Table 4:** **Characteristics of the genomes of *A.sinensis*, *A.gambiae*, *Ae.aegypti*, and *C.quinquefasciatus***

	***A. sinensis***	***A. gambiae***	***Ae. aegypti***	***C. quinquefasciatus***
Genome size (Mbps)	220.8	278	1376	540
Genome coverage (×)	18.8	10.2	7.6	6.1
Number of contig	27,497	18,962	36,206	48,671
Number of scaffold	9,594	8,987	4,758	3,171
Contig N50 (Kbps)	30.1	---	82.6	28.6
Scaffold N50 (Kbps)	814.2	---	1.5	486.8
Average contig size (bps)	7800	13878	36184	---
Average scaffold size (bps)	23012	30930	290873	---
GC (%)	42.6	40.9	38.2	37.4
Number of gene (protein-coding)	16766	12457	15419	18883
Percentage of gene Length (%)	26.4	23.1	17.4	18.5
Percentage of exon region length (%)	11.0	7.2	1.9	4.4

### Repetitive elements analysis

**Figure 1:**
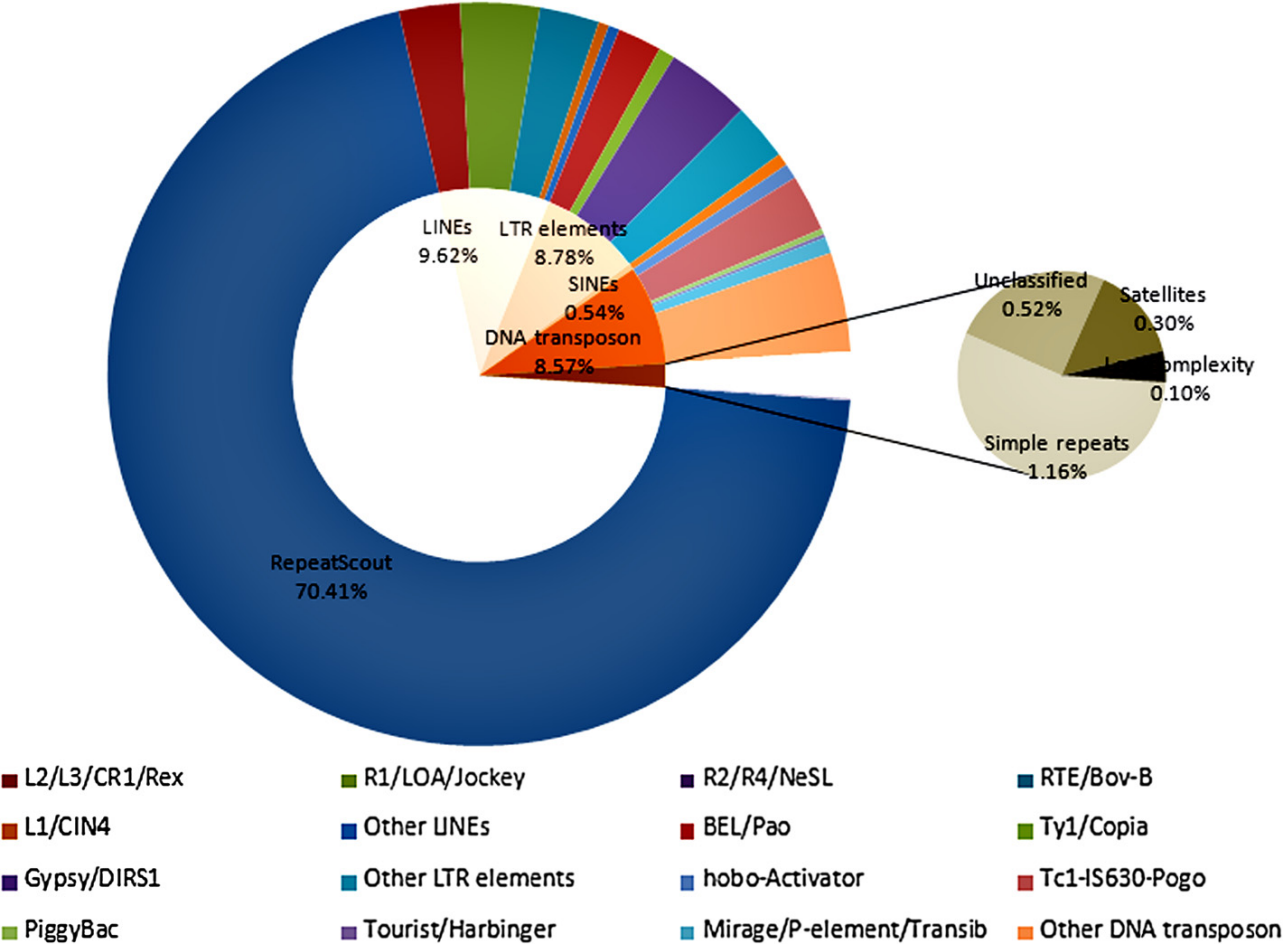
**Repetitive elements in *A. sinensis*.**

We estimated 15,200,821 nt repetitive elements, which accounted for approximately 6% of the *A. sinensis* genome. The most abundant of repetitive elements were transposable elements (TEs) or potential TEs (Figure 1). These constituted about 97.9% of the repetitive elements(70.4% potential TEs and 27.5% TE) and 6% of the genome (Figure 1). Of the remaining repetitive elements, 0.5% were unclassified repeats, 0.3% were satellites, 1.2% were simple repeats and 0.1% were low complexity. In all TEs classification, 19% were retroelements (Class I elements), 9% were DNA transposon elements (Class II elements) and 72% were potential TEs. Class I elements consisted of five clades (*L2/L3/CR1/Rex*, *R1/LOA/Jockey*, *R2/R4/NeSL*, *RTE/Bov-B* and L1/CIN4), while Class II elements also consisted of five clades (*hobo-Activator*, *Tc1-IS630-Pogo*, *PiggyBac*, *Tourist/Harbinger* and *Mirage/P-element/Transib*). Three further clades (*BEL/Pao*, *Ty1/Copia* and *Gypsy/DIRS1*) were identified from long interspersed elements (LINEs) and long terminal repeat (LTR) retrotransposon elements.

Compared with published mosquito genome sequences, the TEs content of *Anophelinae* (*A. gambiae*, 11% to 16%) were far less than *Culicinae* (*Ae. aegypti*, 42% to 47%; *C. quinquefasciatus*, 29%) [21-23]. TEs content could be a leading factor influencing genome size in many species [24,25]. For example, studies have shown that the genome of *Ae. aegypti* has doubled its size as a result of TEs [22]. Thus, the differences in the genome size of *A. sinensis* and other mosquito species could in part be due to the accumulation or loss of TEs in the different species.

### Gene prediction

**Figure 2:**
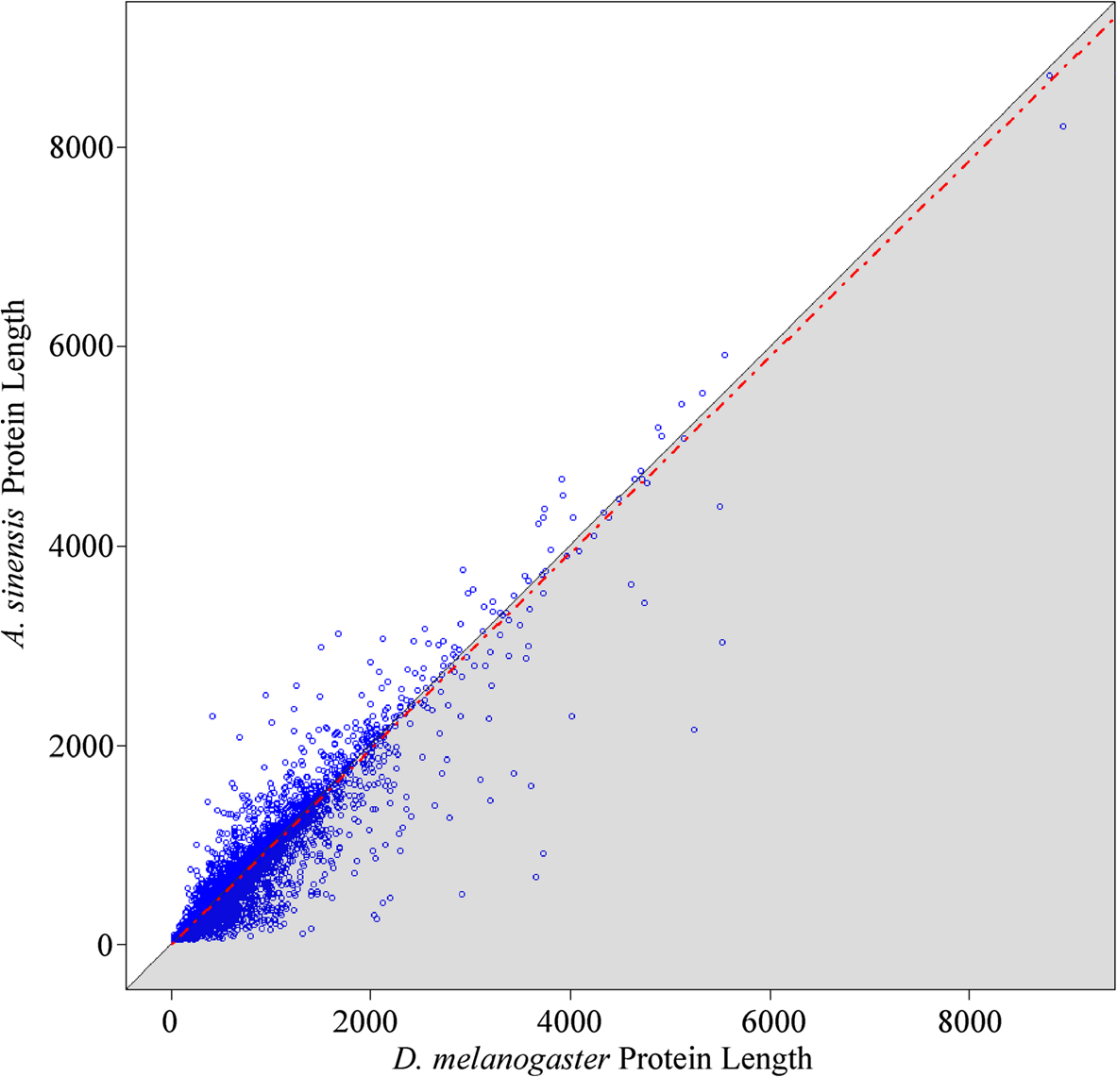
***D. melanogaster *protein length plotted against the orthologous protein length for *A. sinensis*.** The red dashed line shows the results of a linear regression. The concordance of the two variables is presented with 95% confidence intervals. Perfect concordance (1.0) would indicate that all points fall on the line where x = y, depicted by the edge of the grey shading.

Based on homology and *de novo* predictions, we identified 16,766 protein-coding genes with an average transcript length of 2608 bp, a coding sequence size of 1083 bp and 2.9 exons per gene (Additional file 1: Table S4). Given the high conservation of single-copy orthologs, protein lengths should have a high coherence between *A. sinensis* and *D. melanogaster*. We found that *A. sinensis* proteins exhibited slightly lower than expected concordance values with *D. melanogaster* (0.92, see Figure 2), but that this concordance value was similar to that reported for the three other sequenced mosquitoes (*A. gambiae,* 0.92; *Ae. Aegypti,* 0.93; *C. quinquefasciatus*, 0.90) [21-23]. This finding indicates that the gene prediction analysis for *A. sinensis* was robust*.*

Although the predicted genome size of *A. sinensis* was smaller than that of *A. gambiae*, we found a greater number of predicted genes in the genome of *A. sinensis* (Table 4). This discrepancy was consistent with the results of the genome-wide analysis that revealed a higher percentage of exon region length and less TEs content in the genome of *A. sinensis*. Differences in the numbers of predicted genes in these two species may be consequence of species-specific genetic differences that have arisen from biological adaptations to the different environmental challenges faced by these two mosquitoes. However, it is also possible that gene numbers were overestimated in the genome of *A. sinensis* because of false-positive gene predictions. These can arise because of inaccurate annotation of the automated consensus gene set or because of putative TEs and bacterial contaminates which escaped earlier detection. Overestimation of the number of predicted genes has been reported for other mosquito species [23]. The third possibility is genes were under-prediction in *A. gambiae*. It was the second insect genome to be sequenced, and like the initial *D. melanogaster*, the used consortia eliminated most ab initio gene models without comparative or experimental support, which may cause under-prediction. For example, a recent genomic comparative paper on orphan genes in insects does not even include the *A. mellifera* gene set, and notes that the *A. gambiae* gene count is abnormally low for orphan genes [26]. Manual examination of the output will be required to assure the accuracy of the predicted genes of *A. sinensis* found in this study.

In addition to protein-coding genes, we also identified 41 microRNA (miRNA), 348 tRNA and 2017 rRNA genes in the *A. sinensis* genome (Additional file 1: Table S5; see the Additional file 2: “MiRNA list” for a list of all predicted *A. sinensis* miRNA target genes and their annotations). At present, 67 miRNAs have been described for *A. gambiae*, which is almost 1.5 times that found for *A. sinensis*. Our finding of just 41 miRNA for *A. sinensis* might be an underestimation as the target prediction was based on an imperfect match between known miRNA and our genomic sequence. It is also possible that some target genes were missed during the alignment due to the differences between the two genomes of these two anopheline species. Another possible factor that may contribute to lower miRNA genes in *A. sinensis* is that *A. sinensis* may have unidentified miRNAs.

### Functional annotation and gene family analysis

For all predicted protein-coding genes, 93.8% had matches in the non-redundant (NR) databases, 64.6% were similar to entries in the InterPro database, 67.7% were assigned GO terms, 14.2% were mapped to known pathways, 14.0% had signal peptides and 21.4% had transmembrane regions (Additional file 1: Table S6). There were several domains (fibrinogen, protein kinase and six-bladed beta-propeller) and repeats (LDLR class B repeat and Leucine-rich repeat) overrepresented in *A. sinensis* compared to *A. gambiae* (Additional file 1: Table S7). However, no significant differences of PTMs were observed between *A. sinensis* and *A. gambiae*. Several domains (histone, F-box and Zinc finger) were down-represented in anopheline species compared with the culicine species, though no significant differences of translational modification (PTM) and repeat were observed between these two subfamilies (Additional file 1: Table S8).

**Figure 3:**
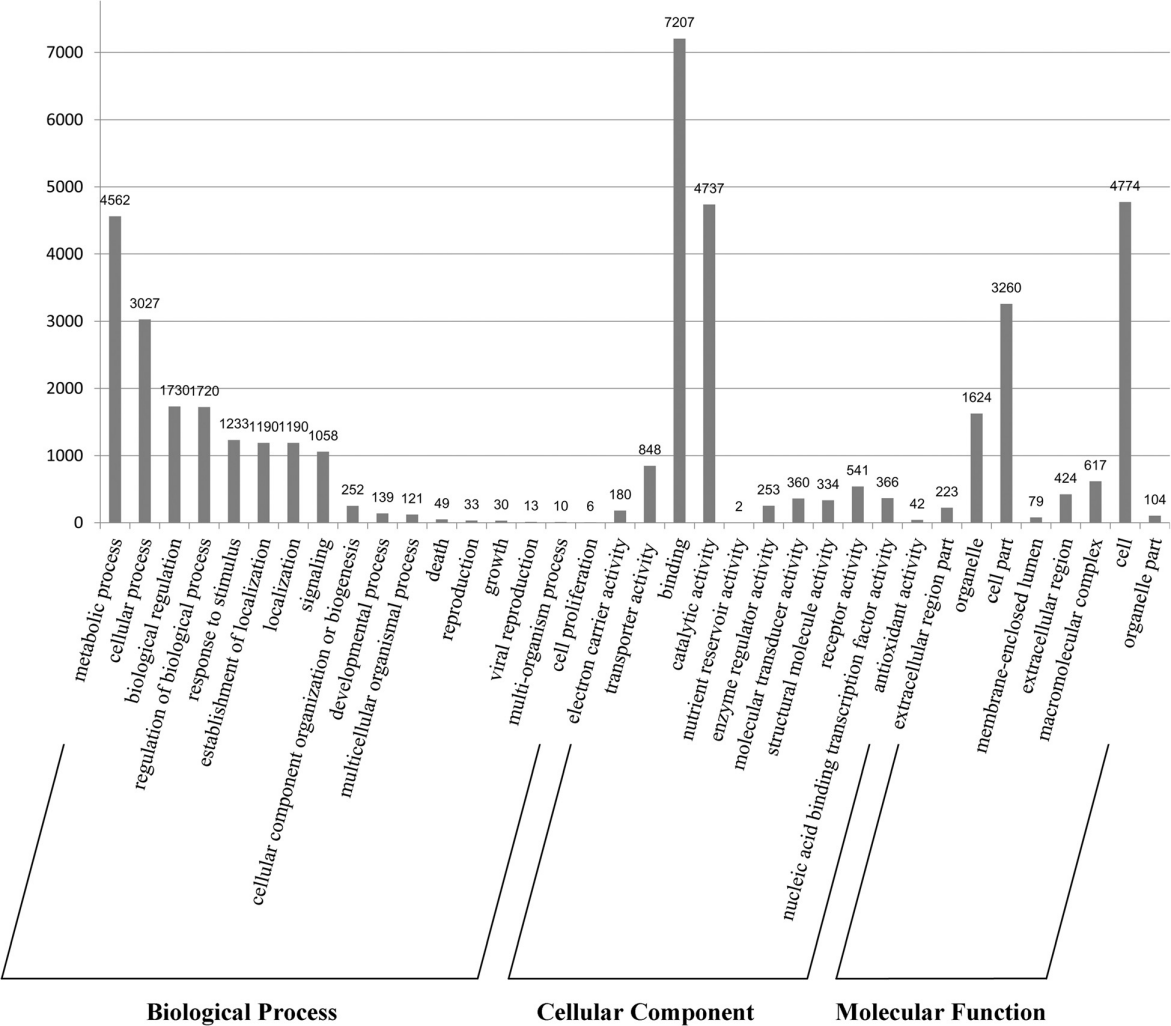
**Gene ontology classifications for *A. sinensis *at level two.** Gene ontology classifications included three components: biological processes, cellular components and molecular functions.

We assessed the functional predictions of proteins according to broad GO categories standardized to level 2 terms (Figure 3). Additional GO analysis of the proteomes revealed differences between *A. sinensis* and *A. gambiae* (Additional file 1: Table S9), and between the anopheline and the culicine species (Additional file 1: Table S10). In the biological process category, proteins involved with signaling processes (GO:0023052) in were expanded in anopheline species compared with the culicine species (Additional file 1: Table S10). While, in the molecular function category, proteins involved in molecular transducer activity (GO:0060089) was expanded in anopheline species compared with culicine species (Additional file 1: Table S10).

Of the entire *A. sinensis* gene set, 2377 genes had an ortholog belonging to one of the 235 known biological pathways. There were no significant differences in the mapping of genes to pathways between *A. sinensis* and *A. gambiae* or between the anopheline and culicine subfamilies.

The frequencies of transmembrane regions in *A. sinensis* were relative lower than other three mosquito species (Additional file 1: Table S11). With just three exceptions (6, 7 and 10), protein numbers tended to decrease with the increasing transmembrane helices (Additional file 1: Table S12). InterPro analysis revealed that olfactory receptors (14.29%), G-protein coupled receptors (GPCRs, 34.91%) and major facilitator superfamily domain (16.25%) accounted for the largest proportion of the predicted proteins of the 6, 7 and 10 transmembrane helices, respectively.

The *A. sinensis* genome revealed 3,972 gene clusters containing 11,300 genes that were common to the genomes of the three previously sequenced mosquito species. There were 4,065 gene clusters containing 10,465 genes in *A. gambiae*, 4,064 gene clusters containing 12,608 genes in *Ae. aegypti*, and 4,073 gene clusters containing 14,827 genes in *C. quinquefasciatus*. 109 clusters found only in the four mosquito genomes, 34 clusters found specific to the *Anophelinae*, and 29 clusters containing 30 genes found specific to *A. sinensis*.

### Gene orthology prediction

**Figure 4:**
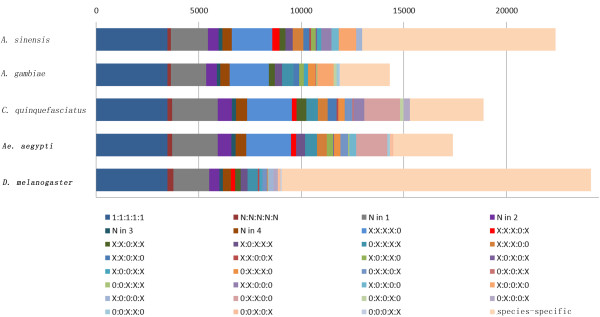
**Ortholog delineation among the protein-coding gene repertoires of the four mosquito species and *D. melanogaster*.** Membership of the categories of orthologous groups are depicted as follows: (i) 1:1:1:1:1 indicates single-copy orthologs in all species; (ii) N:N:N:N:N indicates multi-copy orthologs in all species; (iii) N in 1, N in 2, etc. indicates multi-copy orthologs in one or two species, etc; (iv) x:x:x:x:0, x:0:x:x:x, x:x:0:x:0 etc. indicates (by a 0) which of the five species, in the order listed above, did not contain single-copy or multi-copy orthologs. The remaining proportion of the sequence for each species exhibited no orthologs with genes in the other species (depicted as specific-specific in the figure).

Consistent with evolutionary distance estimates, we observed a higher degree of genetic similarity between *A. sinensis* and other mosquito species proteomes than between *A. sinensis* and *D. melanogaster* proteomes (Figure 4). *A. sinensis* and *A. gambiae* shared the highest number of orthologous genes (48.3%) while *A. sinensis* and *D. melanogaster* shared the lowest number of orthologous genes (36.8%, Figure 4). A total of 4727 orthologous genes were shared only among the mosquitoes.

Analysis of InterPro in these 4727 orthologous genes revealed the most gene-enriched domain and family were peptidase (Additional file 1: Table S13), while analysis of Kyoto Encyclopedia of Genes and Genomes (KEGG) pathway revealed that genes were most enriched in metabolic pathways (Additional file 1: Table S14), both indicating functions central to mosquito biology, such as feeding behavior. Feeding releases peptidase in the midgut and assists in the degradation of blood meal proteins into peptides and amino acids [27].

### Microsynteny with sequenced mosquito genomes

**Figure 5:**
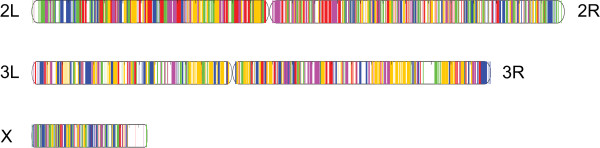
**The coverage of the microsynteny block of *A. sinensis *** **on the chromosome of *A. gambiae*.**

A genome-wide analysis revealed a significantly higher microsynteny between *A. sinensis* and *A. gambiae* (59.8%) than between *A. sinensis* and *Ae. aegypti* (42.1%) or between *A.sinensis* and *C. quinquefasciatus (*39.9%), or *A. sinensis* and *D. melanogaster (*20.4%, Table 5). The largest microsynteny, between *A. sinensis* and *A. gambiae*, also included the most shared gene families (8,457) and the largest coverage of the *A. sinensis* genome (132 M, Table 5). These findings are consistent with our present knowledge of the evolutionary relationship among these species. Given the close relationship between *A. sinensis* and *A. gambiae*, we took the chromosomes of *A. gambiae* as a reference for alignment, and aligned *A. sinensis* to the 2^nd^, 3^rd^ and X chromosomes of *A. gambiae*. Coloring inside the schematic chromosome arms indicated microsynteny matches to a microsynteny block of *A. sinensis* (Figure 5). Chromosomal rearrangements in *A. sinensis* were observed, most obviously with respect to the 2 L chromosome arm of *A. gambiae*. In contrast, chromosomal rearrangements were relatively rare in other chromosomes arms of *A. gambiae* (Additional file 3: Figure. S1). In the genus Drosophila, the interspecies chromosomal rearrangements can be caused by the occurrence of paracentric inversions, Robertsonian translocations or transposon [28]. Such genetic changes may also have contributed to the chromosomal rearrangements observed in *A. sinensis*.

**Table 5:** **Characteristics of microsynteny blocks between *A. sinensis*, *A. gambiae*, *Ae. aegypti*, *C. quinquefasciatus*, and *D. melanogaster***

**Microsynteny blocks**	**Numbers of microsynteny blocks**	**Total length of microsynteny (Mbps)**	**Shared gene families**
*A. sinensis*/*A. gambiae*	927	*A. sinensis*:132	8,457
		*A. gambiae*:157	
*A. sinensis*/*Ae. aegypti*	1,668	*A. sinensis*:93	6,792
		*Ae. aegypti*:398	
*A. sinensis*/*C. quinquefasciatus*	1,690	*A. sinensis*:88	7,087
		*C. quinquefasciatus*:185	
*A. sinensis*/*D. melanogaster*	1,031	*A. sinensis*:45	2,658
		*D. melanogaster*:38	

### Divergence time

**Figure 6:**
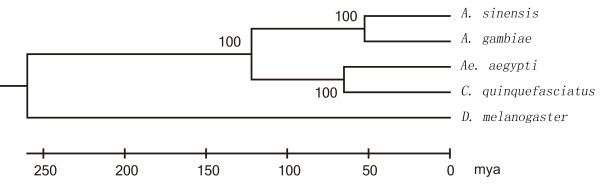
**The inferred supertree for four mosquito species and *D. melanogaster*.** The topology of the supertree was evaluated by bootstrap percentages. Distances are in millions of years.

We calibrated the remaining 2,348 linear trees assuming a divergence time of ~260 million years ago (Mya) between *Drosophila* and *Anopheles*. This is the most rigorously calculated date available for the most recent split involving a mosquito lineage and its sister taxon [29,30]. Based on this basal divergence time, we obtained an estimate of the split between the *Anophelinae* and the *Culicinae* of approximately122 Mya (Figure 6). This is slightly later than a previous estimate of 145-200 Mya, which was inferred from mitochondrial sequences [8]. We estimated the split between *A. sinensis* and *A. gambiae* to have occurred ~52 Mya. This date of divergence was earlier than the split between *A. funestus*, another member in anopheline group, and *A. gambiae* (15–25 Mya) [31].

### Few immune-related gene sets may be associated with malaria vectorial capacity

*Anophelinae* are recognized as major vectors of human malaria, while culicine species are the principal etiological agents of mosquito-borne viruses. It is not surprising that genetic factors play decisive roles in determining vectorial capacity [32]. Previous studies regarding the immune system of *Anophelinae* have shown that changes in certain aspects can affect the development of *Plasmodium* either positively or negatively [33]. As shown in Additional file 1: Table S15, relative to *Culicinae*, C-type lectins (CTLs), serine protease inhibitors (serpins, SRPNs) and MD2-like gene (ML) families have contracted in the *Anophelinae*, whereas the thioester-containing protein (TEP) and peroxidase gene families have expanded, which may result from the differential duplication and/or loss of genes among these evolutionary lineages. Although comparative immune-related gene families in *C. quinquefasciatus*, *Ae. aegypti*, and *A. gambiae* have been studied, limited information is available due to limited numbers of anopheline species. With the discovery of the second anopheline mosquito, *A. sinensis*, we may reveal the *Plasmodium*-susceptible genotype, which will help to understand the details of the relationships between anopheline mosquito vectors and malarial pathogens.

Both the ML and serpin gene families have been shown obviously interfere with malarial infection. *AgMDL1*, an MD2-like receptor, showed specificity in regulating resistance to *P. falciparum* and O’nyong-nyong virus [34,35]. These overlaps between the effects of MLs on *Plasmodium* spp. and other viruses suggest that MLs are a universal defense mechanism for mosquitoes against invading pathogens. Expression of the *SRPN6* gene can limit the number of rodent malarial oocysts in *A. stephensi*[36]*.* Thus, the significant contraction of MLs and Serpins can help the malarial parasite to survive in *Anophelinae*.

The TEP [37] and C-type lectins (CTLs) [38] gene families are both involved in pathogen recognition (PRRs) and immune response activation. One TEP family gene in particular (*TEP1*), can be upregulated after malarial infection and strongly inhibit the development of infection in both rodents and humans by binding to *Plasmodium* parasite surfaces [33]. In contrast, two circulating CTLs from *A. gambiae* (C-type lectin 4 [CTL4] and CTL mannose binding 2 [CTLMA2]) have been identified as agonists of the rodent malaria species, *P. berghei*, which can induce massive ookinete melanization when silenced [39]. Consequently, the downregulation of CTL members and the upregulation of TEP members in *Anophelinae* are likely to depend on their relative roles in promoting or inhibiting the development of malarial parasites. Putative HPX (HPX2, HPX7 and HPX8) can be induced in the mosquitoes midgut in response to *Plasmodium* infection, in order to potentiate nitric oxide toxicity and improve antiplasmodial effects [40,41]. Thus, HPX enzymes have been considered as key enzymes induced in the midgut cells of *A. gambiae* invaded by *Plasmodium* ookinetes.

The observed contraction of these two immune gene families (MLs and serpins) could be explained as important genetic components in the *Plasmodium*-susceptible phenotype. Such gene expression changes occur earlier than the invasion of *Plasmodium*, whereby *Anophelinae* are able to transmit *Plasmodium*. However, *Plasmodium* infection may induce the activation (and subsequent expansion or contraction) of immune-related genes involved in pathogen defense in mosquitoes. The two expanded gene families (TEP and peroxidases) and one contracted gene family (CTLs) observed in this study could have formed gradually as a long-term adaptive immune response against *Plasmodium* infection, and been expanded or contracted under positive selection in *Anophelinae*. Consequently, this immune-related gene set analysis at the theoretical level can provide clues for understanding the genetic basis of a *Plasmodium*-susceptible phenotype. These selective genes may serve as valuable potential targets for future malarial control strategies.

## Methods

### Strain selection and DNA extraction

The laboratory strain of *A. sinensis* used in this study has been inbred within the lab since 1984 and, never been exposed to pesticides. These mosquitoes were reared at 26 ± 1°C and 75 to 85% humidity, under a 10:14 h light:dark cycle. Genomic DNA was extracted from 300 adult females and 300 males (2 to 3 days post adult emergence) according to methods described in [42]. To prevent RNA and protein contamination, extracted DNA was treated with RNase A and proteinase K and, subsequently, precipitated with ethanol.

### Whole-genome sequencing and assembly

We employed a whole-genome sequencing strategy with Roche/454 GS FLX. We constructed a total of five single-end and seven mate-pair sequencing libraries with insert sizes of about 3 Kb, 8 Kb and 20 Kb from 1 μg, 5 μg, 30 μg and 60 μg of starting DNA. In total, we generated 4.16 Gb of data of sequencing reads ranging from 40 to 1196 bp. To reduce the effect of sequencing error during assembly, we undertook a series of checking and filtering steps in assembling the reads generated. By using stringent criteria, 3.34 G of high quality data were incorporated into the final *de novo* genome assembly.

The Lander-Waterman algorithm [43] were used to estimate the genome size of *A. sinensis*. K-mer analysis for single-end reads [44] revealed a frequency distribution that conformed to the Poisson expectation when K-mer was equal to 13. The value of expected depth was calculated based on the lambda, a parameter of possion distribution. The genome size of *A. sinensis* was then calculated using the total K-mer number divided by the expected depth value.

Whole-genome assembly was carried out with a Celera Assembler V6.1 for the remaining 454 reads [45]. The revised pipeline (called Celera Assembler with the Best Overlap Graph, CABOG) was robust to uncertainty in homopolymer run length, high read coverage and heterogeneous read lengths. We utilized the following modules of the Celera Assembler software for successive phases of the assembly: pairwise overlap detection; initial ungapped multiple sequence alignments, called unitigs; unitig consensus calculation; combining unitigs with mate constraints to form contigs and scaffolds that were ungapped and gapped multiple sequence alignments; and, finally, scaffold consensus determination. Because the genome used for sequencing were constructed from whole adult mosquitoes, contamination from bacteria in gut or adhering on the surface were inevitable. To check for possible microbial contamination of the assembly, we screened scaffolds against the NCBI NT database using query alignment and identity cut-off of 90% and e-value cut-off of 1e-6. When the top hit was bacterial species, this scaffold was removed.

In order to assess the assembly quality, the transcriptome was sequenced and aligned to the scaffold sequences using Blat with default parameters [46]. Assembly quality was also assessed by mapping the 454 Single reads to the scaffolds using BWA. The mapped regions (consensus sequences) with depth over 3X were extracted for SNVs and INDEL variation analysis, which represent potential base error and short indel error rate in the genome, respectively [46]. Additionally, presence of CEGs was evaluated for the genome assembly (http://korflab.ucdavis.edu/Datasets/cegma/submit.html) [47,48].

### Identification of repetitive elements

The identification of repetitive elements is essential for genome sequencing, as unidentified repetitive elements can affect the quality of gene predictions, annotation and annotation-dependent analyses [49]. Two methods were adopted for masking repeat regions in *A. sinensis*. First, RepeatMasker V3.3.0 (http://www.repeatmasker.org/) was applied against the Repbase library (species *Anopheles*) based on the scaffolds. Then, RepeatScout V1.0.5 [50] software was used (with frequency set to ≥50) to build a repeat regions database by providing scaffolds and potentially repeat sequences. These results were merged with the results of the transposable elements for mosquitoes, which were downloaded from TEfam database (http://tefam.biochem.vt.edu/tefam/). Finally, these merged results were reprocessed with RepeatMasker.

### Gene prediction

To predict genes, we used two independent approaches: a homology-based method and a *de novo* method. The results of these two methods were integrated by the EVidenceModeler utility and then filtered multiple times and also checked manually. The reference protein sequences for protein alignment were obtained from VectorBase (for the aforementioned three sequenced mosquito species) and the NCBI database (for Culicidae species). CD-HIT software was used to cluster these protein sequences with 100% global similarity [51]. AAT [52] and Genewise [53] software were used to align the protein data to the masked scaffolds. By comparing the databases, we obtained the number of protein distributions.

Four *ab initio* gene prediction programs were run on the genome: SNAP [54], Augustus [55], GlimmerHMM [56], and Genezilla [57] with the model trained using the published mosquito gene information (*A. gambiae, Ae. aegypti* and *C. quinquefasciatus*).

### Quality of protein-coding gene predictions

To estimate the accuracy of gene prediction, we undertook a consistency check for the protein length of single-copy orthologs between *A. sinensis* and *D. melanogaster*. Considering the high conservation of single-copy orthologs, the protein length should have a high coherence between two species [23]. The protein lengths of the two species were plotted as a scatter diagram and analyzed with a regression analysis. We compared the results of this regression analysis with results from the published literature.

### Identification of noncoding RNA genes

tRNA genes were predicted by tRNAscan-SE-1.23 with eukaryote parameters [58]. The rRNA fragments were identified by aligning the rRNA template sequences from the SILVA database [59] and RNAmmer database, by using BlastN at E-value 1e-5 with cutoff of identity ≥95% and match length ≥50 bp. It is important to note that rRNA genes in the *A. sinensis* genome were combined by aligning the 5.8S, 18S, 25S and 28S regions of databases using BlastN. miRNA was predicted by BlastN against the hairpin sequences from miRBase database (RELEASE 17) with E-value 1e-3, allowing no less than 70 bp alignment length, and requiring no less than 85% overall identity and 80% coverage.

### Functional annotation

Gene functions were assigned according to the best match of the alignments using Blast and BlastP (query coverage ≥50%; E-value: 1e-10) against the NCBI NR protein database. All predicted protein-coding genes were obtained with the InterProScan analysis tool [60]. According to features of the predicted protein sequences, the InterProScan analysis was based on the active site, the binding site, the conserved site, the domain, the family, the PTM, and the repeat. Gene Ontology (GO) IDs for each gene were obtained from the corresponding InterProScan entry. All genes were aligned against the KEGG proteins, and the pathway in which the gene might be involved was derived from the matching genes in the KEGG. SignalP 4.0 server was used to predict the presence and location of signal peptide cleavage sites in the amino acid sequences [61]. This method incorporates a prediction of cleavage sites and a signal peptide/non-signal peptide prediction based on a combination of several artificial neural networks. TMHMM software [62] was used with default values to predict the transmembane region based on a hidden Markov model.

### Gene orthology prediction

The gene orthology predictions were generated by the Ensemble Gene Tree method [63], which is based on the PHYML algorithm for multiple protein sequence alignments, and uses MUSCLE for each gene family that contains sequences from all five species (*A. sinensis, A. gambiae, Ae. aegypti, C. quinquefasciatus* and *D. melanogaster*). Gene trees were reconciled with the species trees using the RAL algorithm to call duplication events on internal nodes and to root the trees. The relations of orthology were inferred from the results of each gene tree.

### Defining gene families

The PANTHER hidden Markov models V7.2, annotated to different functional gene families, were used with default parameters (i.e. E-value: 1e-3) to classify all gene models of *A. sinensis*. Immune-related gene sets were downloaded from ImmunoDB resource (http://cegg.unige.ch/Insecta/immunodb) and subjected to inspection, curation, and phylogenetic analysis. Based on these gene sets, we re-annotated the proteins in the *A. sinensis* genome by Blast search, and counted the number of *A. sinensis* genes in each functional gene set. The threshold E-value in the Blast search was set to 1e-3, while the similarity was set to 0.35.

### Construction of microsyntenic blocks

CHSMiner V1.1 [64] was used to construct the microsynteny map for *A. sinensis* and the other three previously sequenced mosquito species. Briefly, the program used the orthologs between two genomes as anchors, and merged two anchors into a block if they were located less than a specified gap size apart. We used default values for parameters and set the minimum length to 100 Kb. Each microsynteny detected was evaluated by corrected *P*-values; only those results with the *P*-values less than 1e-5 were preserved.

### Phylogeny construction

M-Coffee V9.0 program [65] was used to perform the multiple alignment of proteins in each family. A phylogeny tree was constructed based on the 3,470 single-copy families in the five species (*A. sinensis, A. gambiae, Ae. aegypti, C. quinquefasciatus and D. melanogaster*)*.* We used the Phylip package V3.69 [66] to build the maximum likelihood (ML) tree for each protein family under the JTT substitution model. Then the SuperTree software was used to get an integrated supertree. To evaluate the topology of the supertree, we performed a bootstrap resample analysis using 100 resamples from the original tree.

## Conclusions

Malaria is caused by infection with *Plasmodium* parasites that are transmitted via the bites of infected female *Anopheles* mosquitoes. Vector control offers an important means of limiting the spread of malaria; however, the lack of genetic information on *Plasmodium*-susceptible anopheline mosquitoes is a major obstacle to the development of effective vector management. We generated the first draft genome sequence of *Anopheles sinensis*, an Asiatic mosquito species suspected to be the most important vector of *P. vivax*. We compared the genetic composition of this species to that of other sequenced mosquito species in the subfamily *Anophelinae* and the subfamily *Culicinae* (the latter are not susceptible to *Plasmodium* infection). The results of these comparisons provide important genetic insights into this vector-disease system. In particular,we observed the expansion and contraction of several important immune-related gene families known to influence aspects of *Plasmodium* development, in the anopheline species relative to the culicine species. These differences suggest that species-specific immune responses to *Plasmodium* infection underpin the biological differences in *Plasmodium* susceptibility that characterize these two mosquito subfamilies. This study provides critical genomic information that will pave the way for analyses investigating the genetic basis of mosquito susceptibility and resistance to *Plasmodium* parasites.

## Competing interests

The authors declare that they have no competing interests.

## Authors’ contributions

BS and CLZ are the principal investigators and project managers in this work. HYZ and CRX conducted the sample collection. DZ, LNS and BS extracted DNA. DZ, YX and QH edited tables and figures. DZ, GHD, BS, CLZ, DHZ, SDL, SCH, XYY, PZ, CC, XLC, WJW, YL, YTY, YS, LM did the genomics analysis. DZ, DHZ, JY, and BS wrote and edited the manuscript. All authors read and approved the final manuscript.

## Supplementary Material

Additional file 1: Table S1 Putative contaminating scaffold sequence of possible bacterial origin. **Table S2:** Assessment of gene coverage by assembled transcripts of *A. sinensis. ***Table S3:** Variation statistics regarding mapping of raw reads to the scaffolds. **Table S4:** Results of gene prediction and predicted protein-coding genes for *A. sinensis. ***Table S5:** Identification of non-coding RNA genes in the *A. sinensis* genome. **Table S6:** Functional annotation of predicted genes for *A. sinensis.* **Table S7:** Occurrence of the over-represented InterPro domains and repeats in the genome of *A. sinensis* compared with the genome of *A.gambiae*. **Table S8:** Occurrence of the down-represented InterPro domains in the genomes of the Anopheline species compared with the Culicine species. **Table S9:**  Occurrence of the over-represented level 2 GO terms in the genome of *A. sinensis* compared with the genome of *A.gambiae*. **Table S10:** Occurrence of the over-represented level 2 GO terms in the genomes of the Anopheline species compared with the Culicine species. **Table S11:** Number of transmembrane regions in the four mosquito species. **Table S12:** Distribution results of the transmembrane regions in the four mosquito species. **Table S13:** Occurrence of the top 10 domains and families enriched in orthologous genes that were shared only among the four mosquito species. **Table S14:** Occurrence of the top 12 pathways enriched in orthologous genes that were shared only among the four mosquito species. **Table S15:** Number of selected immune-related gene sets in the four mosquito species. Click here for file

Additional file 2 **MiRNA list of all predicted *A. sinensis *** **miRNA target genes and their annotations.** Click here for file

Additional file 3: Figure S1 Microsynteny between the genomes of *A. sinensis* and *A. gambiae*. Genomic scaffolds of *A. sinensis* inferred to be syntenic were linked (blue lines) to *A. gambiae* chromosomes. Click here for file
