# Supplementary material for: Genome sequence of Anopheles sinensis provides insight into genetics basis of mosquito competence for malaria parasites
Source: BMC Genomics. 2014 Jan 18;15:42. doi: 10.1186/1471-2164-15-42 (PMC3901762; doi:10.1186/1471-2164-15-42)
Supplement: Additional file 1: Table S1 — Putative contaminating scaffold sequence of possible bacterial origin. Table S2: Assessment of gene coverage by assembled transcripts of A. sinensis. Table S3: Variation statistics regarding mapping of raw reads to the scaffolds. Table S4: Results of gene prediction and predicted protein-coding genes for A. sinensis. Table S5: Identification of non-coding RNA genes in the A. sinensis genome. Table S6: Functional annotation of predicted genes for A. sinensis. Table S7: Occurrence of the over-represented InterPro domains and repeats in the genome of A. sinensis compared with the genome of A.gambiae. Table S8: Occurrence of the down-represented InterPro domains in the genomes of the Anopheline species compared with the Culicine species. Table S9: Occurrence of the over-represented level 2 GO terms in the genome of A. sinensis compared with the genome of A.gambiae. Table S10: Occurrence of the over-represented level 2 GO terms in the genomes of the Anopheline species compared with the Culicine species. Table S11: Number of transmembrane regions in the four mosquito species. Table S12: Distribution results of the transmembrane regions in the four mosquito species. Table S13: Occurrence of the top 10 domains and families enriched in orthologous genes that were shared only among the four mosquito species. Table S14: Occurrence of the top 12 pathways enriched in orthologous genes that were shared only among the four mosquito species. Table S15: Number of selected immune-related gene sets in the four mosquito species. [file 1471-2164-15-42-S1.doc]

Table S1: Putative contaminating scaffold sequence of possible bacterial origin.

| Scaffold | Query length (bps) | Subject length (bps) | Query alignment | Annotation | E-value | Identity |
| --- | --- | --- | --- | --- | --- | --- |
| scf7180000689203 | 3419 | 3944163 | 90% | AM889285.1 | 0 | 93% |
| scf7180000689880 | 1087 | 10657107 | 100% | CP002994.1 | 0 | 94% |
| scf7180000689881 | 1245 | 1032 | 98% | embFR718667.1 | 0 | 98% |

Table S2: Assessment of gene coverage by assembled transcripts of *A. sinensis*

|  | Number（isotig+singleton） | With >90% Sequence in Scaffold | | With >50% Sequence in Scaffold | |
| --- | --- | --- | --- | --- | --- |
|  |  | Number | Percent (%) | Number | Percent (%) |
| transcript | 33010 | 30504 | 92.4 | 31933 | 96.7 |

Table S3: Variation statistics regarding mapping of raw reads to the scaffolds

|  | Homozygous | Heterozygous | Total |
| --- | --- | --- | --- |
| SNVs | 32541 | 386964 | 419505 |
| INDELs | 24615 | 95976 | 120591 |

Table S4: Results of gene prediction and predicted protein-coding genes for *A. sinensis.*

| Gene set | Number | Average transcript length (bp) | Average CDS length (bp) | Average exons per gene | Average exon length (bp) | Average intron length (bp) |
| --- | --- | --- | --- | --- | --- | --- |
| *An. sinensis* | 16766 | 2608. | 1083 | 2.9 | 374 | 802 |
| *An. gambiae* | 10576 | 6325. | 1671 | 4.4 | 430 | 1305. |
| *Ae. aegypti* | 11382 | 17699. | 1518 | 4.3 | 412. | 4812 |
| *Cx. quinquefasciatus* | 11741 | 6493 | 1466 | 4.0 | 374. | 1655 |
| *D. melanogaster* | 4910 | 6956 | 1511 | 4.0 | 493. | 1665. |

Table S5: Identification of non-coding RNA genes in the *A. sinensis* genome

| Type |  | Counts in genome | Average length (bp) | Total length (bp) | % of genome |
| --- | --- | --- | --- | --- | --- |
| miRNA |  | 56 | 85.4 | 4,783 | 0.0021 |
| tRNA |  | 348 | 79.2 | 27,572 | 0.0125 |
| rRNA | 18S | 939 | 88.5 | 83,108 | 0.0376 |
|  | 28S | 1052 | 174.3 | 183,320 | 0.0830 |
|  | 5.8S | 9 | 152.8 | 1,375 | 0.0006 |
|  | 5S | 17 | 108.3 | 1,841 | 0.0008 |
| Total |  | 2017 | 133.7 | 269,644 | 0.1220 |

Table S6: Functional annotation of predicted genes for *A. sinensis.*

| Reference Database | Number (16766) | Percent (%) |
| --- | --- | --- |
| NR | 15727 | 93.8 |
| InterPro | 10826 | 64.6 |
| GO | 11344 | 67.7 |
| KEGG | 2377 | 14.2 |
| Signal peptide | 2341 | 14.0 |
| Transmembrane regions | 3579 | 21.4 |

Table S7: Occurrence of the over-represented InterPro domains and repeats in the genome of *A. sinensis* compared with the genome of *A.gambiae*.

| InterPro type | InterPro ID | *A. sinensis* | *A. gambiae* | Description |
| --- | --- | --- | --- | --- |
| Domain | IPR020635 | 230 | 41 | Tyrosine-protein kinase, catalytic domain |
| Domain | IPR014716 | 178 | 48 | Fibrinogen, alpha/beta/gamma chain, C-terminal globular, subdomain 1 |
| Domain | IPR002181 | 179 | 49 | Fibrinogen, alpha/beta/gamma chain, C-terminal globular |
| Domain | IPR014715 | 137 | 41 | Fibrinogen, alpha/beta/gamma chain, C-terminal globular, subdomain 2 |
| Domain | IPR002290 | 249 | 128 | Serine/threonine-protein kinase, catalytic domain |
| Domain | IPR003595 | 23 | 1 | Protein-tyrosine phosphatase, catalytic |
| Domain | IPR011042 | 89 | 47 | Six-bladed beta-propeller, TolB-like |
| Domain | IPR001245 | 97 | 55 | Serine-threonine/tyrosine-protein kinase |
| Repeat | IPR000033 | 50 | 10 | LDLR class B repeat |
| Repeat | IPR003591 | 148 | 96 | Leucine-rich repeat, typical subtype |

Table S8: Occurrence of the down-represented InterPro domains in the genomes of the Anopheline species compared with the Culicine species.

| InterPro type | InterPro ID | *A. sinensis* | *A. gambiae* | *Ae. aegypti* | *C. quinquefasciatus* | Description |
| --- | --- | --- | --- | --- | --- | --- |
| Domain | IPR007125 | 11 | 37 | 65 | 103 | Histone core |
| Domain | IPR009072 | 31 | 56 | 86 | 124 | Histone-fold |
| Domain | IPR015880 | 296 | 384 | 719 | 803 | Zinc finger, C2H2-like |
| Domain | IPR022364 | 31 | 27 | 38 | 129 | F-box domain, Skp2-like |
| Domain | IPR013087 | 250 | 292 | 580 | 635 | Zinc finger, C2H2-type/integrase, DNA-binding |
| Domain | IPR006612 | 12 | 16 | 53 | 62 | Zinc finger, C2CH-type |
| Domain | IPR001810 | 35 | 34 | 42 | 139 | F-box domain, cyclin-like |
| Domain | IPR007588 | 17 | 21 | 67 | 57 | Zinc finger, FLYWCH-type |
| Domain | IPR001878 | 24 | 24 | 60 | 82 | Zinc finger, CCHC-type |
| Domain | IPR007087 | 330 | 366 | 695 | 755 | Zinc finger, C2H2 |
| Domain | IPR012934 | 97 | 104 | 283 | 349 | Zinc finger, AD-type |

Table S9: Occurrence of the over-represented level 2 GO terms in the genome of *A. sinensis* compared with the genome of *A.gambiae*.

.

| GO category | GO level | GO ID | *A. sinensis* | *A. gambiae* | Description |
| --- | --- | --- | --- | --- | --- |
| Biological process | 2 | GO:0008152 | 4562 | 3755 | Metabolic process |
| Biological process | 2 | GO:0009987 | 3027 | 2502 | Cellular process |
| Biological process | 2 | GO:0016032 | 13 | 0 | Viral reproduction |
| Biological process | 2 | GO:0050789 | 1720 | 1399 | Regulation of biological process |
| Biological process | 2 | GO:0065007 | 1730 | 1409 | Biological regulation |
| Biological process | 2 | GO:0051704 | 10 | 0 | Multi-organism process |
| Biological process | 2 | GO:0050896 | 1233 | 986 | Response to stimulus |
| Cellular component | 2 | GO:0044421 | 223 | 82 | Extracellular region part |
| Cellular component | 2 | GO:0005576 | 424 | 260 | Extracellular region |

Table S10: Occurrence of the over-represented level 2 GO terms in the genomes of the Anopheline species compared with the Culicine species.

.

| GO category | GO level | GO ID | *A. sinensis* | *A. gambiae* | *Ae. aegypti* | *C. quinquefasciatus* | Description |
| --- | --- | --- | --- | --- | --- | --- | --- |
| Biological process | 2 | GO:0023052 | 1058 | 860 | 826 | 842 | Signaling |
| Molecular function | 2 | GO:0060089 | 360 | 353 | 322 | 296 | Molecular transducer activity |

Table S11: Number of transmembrane regions in the four mosquito species.

| Species | Transmembrane region |
| --- | --- |
| *A. sinensis* | 3579 |
| *A. gambiae* | 3504 |
| *Ae. aegypti* | 3851 |
| *C. quinquefasciatus* | 3697 |

Table S12: Distribution results of the transmembrane regions in the four mosquito species.

| #TMHs | *A. sinensis* | *A. gambiae* | *Ae. aegypti* | *C. quinquefasciatus* |
| --- | --- | --- | --- | --- |
| 1 | 1874 | 1534 | 1694 | 1684 |
| 2 | 433 | 446 | 502 | 487 |
| 3 | 224 | 190 | 247 | 254 |
| 4 | 198 | 249 | 264 | 262 |
| 5 | 120 | 155 | 155 | 166 |
| 6 | 161 | 185 | 212 | 190 |
| 7 | 169 | 226 | 230 | 179 |
| 8 | 55 | 95 | 98 | 80 |
| 9 | 58 | 70 | 72 | 79 |
| 10 | 80 | 88 | 89 | 79 |
| 10+ | 234 | 266 | 288 | 237 |

TMHs: transmembrane helices

Table S13: Occurrence of the top 10 domains and families enriched in orthologous genes that were shared only among the four mosquito species.

| InterPro type | InterPro ID | Protein numbers | Description |
| --- | --- | --- | --- |
| Domain | IPR009003 | 231 | Peptidase cysteine/serine, trypsin-like |
| Domain | IPR001254 | 229 | Peptidase S1/S6, chymotrypsin/Hap |
| Domain | IPR007087 | 145 | Zinc finger, C2H2 |
| Domain | IPR015880 | 137 | Zinc finger, C2H2-like |
| Domain | IPR013087 | 124 | Zinc finger, C2H2-type/integrase, DNA-binding |
| Domain | IPR013783 | 70 | Immunoglobulin-like fold |
| Domain | IPR012934 | 67 | Zinc finger, AD-type |
| Domain | IPR002557 | 62 | Chitin binding domain |
| Domain | IPR007110 | 57 | Immunoglobulin-like |
| Domain | IPR011009 | 56 | Protein kinase-like domain |
| Domain | IPR013083 | 49 | Zinc finger, RING/FYVE/PHD-type |
| Family | IPR001314 | 170 | Peptidase S1A, chymotrypsin-type |
| Family | IPR000618 | 74 | Insect cuticle protein |
| Family | IPR001128 | 71 | Cytochrome P450 |
| Family | IPR002401 | 49 | Cytochrome P450, E-class, group I |
| Family | IPR013604 | 40 | 7TM chemoreceptor |
| Family | IPR004117 | 37 | Olfactory receptor, Drosophila |
| Family | IPR006170 | 33 | Pheromone/general odorant binding protein, PBP/GOBP |
| Family | IPR000276 | 32 | GPCR, rhodopsin-like, 7TM |
| Family | IPR010512 | 31 | Protein of unknown function DUF1091 |
| Family | IPR001071 | 25 | Cellular retinaldehyde binding/alpha-tocopherol transport |
| Family | IPR005828 | 23 | General substrate transporter |

Table S14: Occurrence of the top 12 pathways enriched in orthologous genes that were shared only among the four mosquito species.

| Pathway ID | KO numbers | Protein numbers | Description |
| --- | --- | --- | --- |
| ko01100 | 47 | 61 | Metabolic pathways |
| ko01110 | 15 | 21 | Biosynthesis of secondary metabolites |
| ko04010 | 13 | 15 | MAPK signaling pathway |
| ko04510 | 12 | 15 | Focal adhesion |
| ko01120 | 11 | 15 | Microbial metabolism in diverse environments |
| ko04141 | 10 | 10 | Protein processing in endoplasmic reticulum |
| ko04725 | 10 | 11 | Cholinergic synapse |
| ko04810 | 9 | 11 | Regulation of actin cytoskeleton |
| ko04062 | 9 | 10 | Chemokine signaling pathway |
| ko04142 | 9 | 10 | Lysosome |
| ko04912 | 9 | 10 | GnRH signaling pathway |
| ko04910 | 9 | 10 | Insulin signaling pathway |

Table S15: Number of selected immune-related gene sets in the four mosquito species.

| immune-related gene sets | *A. sinensis* | *A. gambiae* | *Ae. aegypti* | *C. quinquefasciatus* |
| --- | --- | --- | --- | --- |
| Caspases | 11 | 14 | 11 | 16 |
| C-type lectins | 24 | 24 | 39 | 55 |
| Galactoside-binding lectins | 8 | 10 | 12 | 11 |
| Peroxidases |  |  |  |  |
| Glutathione peroxidases | 3 | 3 | 3 | 3 |
| Heme peroxidases | 16 | 18 | 12 | 11 |
| Thioredoxin peroxidases | 5 | 5 | 5 | 5 |
| Inhibitors of apoptosis | 6 | 8 | 5 | 6 |
| IMD pathway members | 12 | 7 | 9 | 8 |
| Signal transduction | 3 | 4 | 3 | 5 |
| Lysozymes | 2 | 8 | 7 | 4 |
| MD2-like receptors | 15 | 15 | 26 | 19 |
| Peptidoglycan recognition proteins | 8 | 11 | 8 | 10 |
| Relish-like proteins | 2 | 2 | 3 | 3 |
| Superoxide dismutatses | 6 | 5 | 6 | 5 |
| Spaetzle-like proteins | 6 | 6 | 9 | 7 |
| Serine Protease Inhibitors | 19 | 21 | 29 | 42 |
| Thio-ester containing proteins | 12 | 13 | 8 | 10 |
| Toll-receptors | 10 | 10 | 12 | 9 |
| Toll pathway members | 6 | 5 | 5 | 5 |
