# Supplementary material for: Genome sequence of Anopheles sinensis provides insight into genetics basis of mosquito competence for malaria parasites
Source: BMC Genomics. 2014 Jan 18;15:42. doi: 10.1186/1471-2164-15-42 (PMC3901762; doi:10.1186/1471-2164-15-42)
Supplement: Additional file 2 — MiRNA list of all predicted A. sinensis miRNA target genes and their annotations. [file 1471-2164-15-42-S2.pdf]

miRNAName	TargetGeneID	TargetGeneCount
aga-miR-2-2:[1782337_1782416];	evm.TU.scf7180000695763.34;evm.TU.scf7180000696131.96;evm.TU.scf7180000695680.40;evm.TU.scf7180000695763.23;evm.TU.scf7180000695805.8;evm.TU.scf7180000695763.115;evm.TU.scf7180000696056.99;evm.TU.scf7180000695763.81;evm.TU.scf7180000695805.5;evm.TU.scf7180000695763.119;evm.TU.scf7180000695763.123;evm.TU.scf7180000695763.65;evm.TU.scf7180000695763.8;evm.TU.scf7180000695549.87;evm.TU.scf7180000696060.200;evm.TU.scf7180000695787.101;evm.TU.scf7180000695763.111;evm.TU.scf7180000695763.17;evm.TU.scf7180000695763.25;evm.TU.scf7180000696055.460;evm.TU.scf7180000695763.76;evm.TU.scf7180000695976.29;evm.TU.scf7180000695763.70;evm.TU.scf7180000695763.142;evm.TU.scf7180000695763.89;evm.TU.scf7180000696088.4;evm.TU.scf7180000695763.153;evm.TU.scf7180000696131.269;evm.TU.scf7180000695763.38;evm.TU.scf7180000696071.4;evm.TU.scf7180000695763.11;evm.TU.scf7180000695763.109;evm.TU.scf7180000695763.91;evm.TU.scf7180000695551.16;evm.TU.scf7180000690932.1;evm.TU.scf7180000695763.117;evm.TU.scf7180000695763.55;evm.TU.scf7180000695763.15;evm.TU.scf7180000	198

aga-miR-1:[1035733_1035815];cqu-mir-1:[1035741_1035815];dwi-mir-1:[1035746_1035817];dya-mir-1:[1035746_1035821];	evm.TU.scf7180000691397.9;evm.TU.scf7180000695557.94;evm.TU.scf7180000691397.3;evm.TU.scf7180000695645.2;evm.TU.scf7180000695555.21;evm.TU.scf7180000688107.1;evm.TU.scf7180000696011.24;evm.TU.scf7180000694604.1;evm.TU.scf7180000696025.56;evm.TU.scf7180000695865.9;evm.TU.scf7180000696023.46;evm.TU.scf7180000696060.301;evm.TU.scf7180000695747.27;evm.TU.scf7180000695705.15;evm.TU.scf7180000696025.80;evm.TU.scf7180000695516.14;evm.TU.scf7180000696034.44;evm.TU.scf7180000695882.47;evm.TU.scf7180000696133.36;evm.TU.scf7180000695681.47;evm.TU.scf7180000695653.109;evm.TU.scf7180000695804.2;evm.TU.scf7180000696025.107;evm.TU.scf7180000696025.63;evm.TU.scf7180000695979.1;evm.TU.scf7180000696025.12;evm.TU.scf7180000695929.3;evm.TU.scf7180000696038.1;evm.TU.scf7180000696135.129;evm.TU.scf7180000695648.24;evm.TU.scf7180000696054.13;evm.TU.scf7180000696057.89;evm.TU.scf7180000687438.9;evm.TU.scf7180000695556.155;evm.TU.scf7180000696025.71;evm.TU.scf7180000695562.5;evm.TU.scf7180000695865.225;evm.TU.scf7180000696053.90;evm.TU.scf7180000691397.	207

aga-miR-92b:[64014_64089];	evm.TU.scf7180000696075.5;evm.TU.scf7180000695985.2;evm.TU.scf7180000695705.158;evm.TU.scf7180000696075.13;evm.TU.scf7180000696053.51;evm.TU.scf7180000695709.13;evm.TU.scf7180000696113.8;evm.TU.scf7180000695680.17;evm.TU.scf7180000696065.22;evm.TU.scf7180000696075.12;evm.TU.scf7180000696049.170;evm.TU.scf7180000696135.189;evm.TU.scf7180000696075.11;evm.TU.scf7180000695048.10;evm.TU.scf7180000695705.334;evm.TU.scf7180000696007.50;evm.TU.scf7180000695709.132;evm.TU.scf7180000690005.37;evm.TU.scf7180000695569.1;evm.TU.scf7180000695709.74;evm.TU.scf7180000696056.12;evm.TU.scf7180000696039.269;evm.TU.scf7180000696050.134;evm.TU.scf7180000695712.10;evm.TU.scf7180000696116.64;evm.TU.scf7180000686969.1;evm.TU.scf7180000696056.204;evm.TU.scf7180000695705.198;evm.TU.scf7180000696057.232;evm.TU.scf7180000696050.160	30

aga-miR-219:[431428_431507];aae-mir-219:[431428_431510];	evm.TU.scf7180000695483.87;evm.TU.scf7180000695780.21;evm.TU.scf7180000696015.61;evm.TU.scf7180000696023.50;evm.TU.scf7180000696023.116;evm.TU.scf7180000689293.4;evm.TU.scf7180000696003.31;evm.TU.scf7180000696010.12;evm.TU.scf7180000694985.40;evm.TU.scf7180000690005.68;evm.TU.scf7180000696023.23;evm.TU.scf7180000696101.2;evm.TU.scf7180000696023.41;evm.TU.scf7180000696023.61;evm.TU.scf7180000696038.1;evm.TU.scf7180000696079.29;evm.TU.scf7180000696023.3;evm.TU.scf7180000696023.36;evm.TU.scf7180000695491.41;evm.TU.scf7180000696025.60;evm.TU.scf7180000696023.84;evm.TU.scf7180000695742.49;evm.TU.scf7180000696023.29;evm.TU.scf7180000696023.91;evm.TU.scf7180000696023.82;evm.TU.scf7180000696023.16;evm.TU.scf7180000695557.151;evm.TU.scf7180000695491.61;evm.TU.scf7180000696023.71;evm.TU.scf7180000696055.631;evm.TU.scf7180000695920.21;evm.TU.scf7180000695893.24;evm.TU.scf7180000696013.66;evm.TU.scf7180000688244.7;evm.TU.scf7180000696029.41;evm.TU.scf7180000695675.2;evm.TU.scf7180000696052.10;evm.TU.scf7180000696023.115;evm.TU.scf7180000695935	141

aga-miR-282:[323782_323866];	evm.TU.scf7180000696050.195;evm.TU.scf7180000696060.202;evm.TU.scf7180000695988.5;evm.TU.scf7180000695988.12;evm.TU.scf7180000689694.17;evm.TU.scf7180000695988.4;evm.TU.scf7180000695341.1;evm.TU.scf7180000689180.55;evm.TU.scf7180000696047.81;evm.TU.scf7180000695690.25;evm.TU.scf7180000696131.255;evm.TU.scf7180000695970.52;evm.TU.scf7180000696056.124;evm.TU.scf7180000695988.14;evm.TU.scf7180000695684.32;evm.TU.scf7180000696057.135;evm.TU.scf7180000696060.312;evm.TU.scf7180000696022.21;evm.TU.scf7180000696130.70;evm.TU.scf7180000696055.591;evm.TU.scf7180000695549.42;evm.TU.scf7180000695988.11;evm.TU.scf7180000696048.146;evm.TU.scf7180000694956.12;evm.TU.scf7180000696013.24;evm.TU.scf7180000695971.91;evm.TU.scf7180000695016.1;evm.TU.scf7180000695780.18;evm.TU.scf7180000696054.22;evm.TU.scf7180000696013.87;evm.TU.scf7180000695557.93;evm.TU.scf7180000696039.177;evm.TU.scf7180000696055.384;evm.TU.scf7180000695557.5;evm.TU.scf7180000695709.131;evm.TU.scf7180000695750.17;evm.TU.scf7180000696056.40;evm.TU.scf7180000696106.49;evm.TU.scf71800	71

hsa-miR-4683:[110273_110350];	evm.TU.scf7180000695970.125;evm.TU.scf7180000695681.361;evm.TU.scf7180000689033.1;evm.TU.scf7180000696070.15;evm.TU.scf7180000695653.120;evm.TU.scf7180000696011.71;evm.TU.scf7180000695822.1;evm.TU.scf7180000696057.86;evm.TU.scf7180000696130.71;evm.TU.scf7180000696045.111;evm.TU.scf7180000696131.169;evm.TU.scf7180000696056.1;evm.TU.scf7180000695490.3;evm.TU.scf7180000695555.20;evm.TU.scf7180000696006.50;evm.TU.scf7180000696118.64;evm.TU.scf7180000695794.3;evm.TU.scf7180000696055.220;evm.TU.scf7180000695980.63;evm.TU.scf7180000695947.39;evm.TU.scf7180000696050.183;evm.TU.scf7180000696120.24;evm.TU.scf7180000695970.163;evm.TU.scf7180000696104.3;evm.TU.scf7180000695893.4;evm.TU.scf7180000696052.33;evm.TU.scf7180000695763.5;evm.TU.scf7180000695676.21;evm.TU.scf7180000695981.30;evm.TU.scf7180000696028.28;evm.TU.scf7180000695980.95;evm.TU.scf7180000695747.11;evm.TU.scf7180000696126.39;evm.TU.scf7180000690376.1;evm.TU.scf7180000696007.36;evm.TU.scf7180000696060.350;evm.TU.scf7180000695921.5;evm.TU.scf7180000696041.109;evm.TU.scf7180000696	79

aga-miR-8:[277396_277481];cqu-mir-8:[277400_277477];nlo-mir-8:[277401_277474];dya-mir-8:[277401_277476];	evm.TU.scf7180000695900.33;evm.TU.scf7180000696110.28;evm.TU.scf7180000695751.1;evm.TU.scf7180000696035.69;evm.TU.scf7180000695695.9;evm.TU.scf7180000695988.5;evm.TU.scf7180000695988.12;evm.TU.scf7180000696012.63;evm.TU.scf7180000690427.6;evm.TU.scf7180000695707.23;evm.TU.scf7180000696055.442;evm.TU.scf7180000696069.17;evm.TU.scf7180000695708.33;evm.TU.scf7180000695787.71;evm.TU.scf7180000696133.79;evm.TU.scf7180000695747.36;evm.TU.scf7180000696045.32;evm.TU.scf7180000696010.48;evm.TU.scf7180000696013.35;evm.TU.scf7180000695738.1;evm.TU.scf7180000695236.35;evm.TU.scf7180000696079.5;evm.TU.scf7180000695900.31;evm.TU.scf7180000696108.19;evm.TU.scf7180000696135.129;evm.TU.scf7180000695471.8;evm.TU.scf7180000696045.99;evm.TU.scf7180000696056.210;evm.TU.scf7180000696051.151;evm.TU.scf7180000695707.6;evm.TU.scf7180000695604.8;evm.TU.scf7180000696108.6;evm.TU.scf7180000696108.16;evm.TU.scf7180000696055.13;evm.TU.scf7180000696051.103;evm.TU.scf7180000695707.4;evm.TU.scf7180000695695.14;evm.TU.scf7180000695556.75;evm.TU.scf7180000695707.	214

cqu-miR-14:[338796_338892];aga-mir-14:[338810_338893];	evm.TU.scf7180000696003.25;evm.TU.scf7180000696055.504;evm.TU.scf7180000696116.58;evm.TU.scf7180000689694.17;evm.TU.scf7180000695787.60;evm.TU.scf7180000696003.23;evm.TU.scf7180000695763.87;evm.TU.scf7180000696003.37;evm.TU.scf7180000695543.22;evm.TU.scf7180000695956.31;evm.TU.scf7180000695719.21;evm.TU.scf7180000694922.9;evm.TU.scf7180000696003.40;evm.TU.scf7180000696003.8;evm.TU.scf7180000696032.66;evm.TU.scf7180000695806.25;evm.TU.scf7180000695526.17;evm.TU.scf7180000695702.8;evm.TU.scf7180000695690.15;evm.TU.scf7180000695627.6;evm.TU.scf7180000696077.24;evm.TU.scf7180000695939.80;evm.TU.scf7180000695719.25;evm.TU.scf7180000696005.36;evm.TU.scf7180000696003.51;evm.TU.scf7180000696003.12;evm.TU.scf7180000695660.4;evm.TU.scf7180000696073.12;evm.TU.scf7180000694950.1;evm.TU.scf7180000696060.299;evm.TU.scf7180000696025.60;evm.TU.scf7180000696094.1;evm.TU.scf7180000696126.39;evm.TU.scf7180000696055.249;evm.TU.scf7180000696003.26;evm.TU.scf7180000695608.39;evm.TU.scf7180000693062.1;evm.TU.scf7180000696015.39;evm.TU.scf7180000696003.53	114

aga-miR-137:[771419_771507];cqu-mir-137:[771421_771506];tca-mir-137:[771428_771502];api-mir-137:[771428_771506];	evm.TU.scf7180000694302.4;evm.TU.scf7180000688324.1;evm.TU.scf7180000695652.23;evm.TU.scf7180000695611.30;evm.TU.scf7180000695941.16;evm.TU.scf7180000696023.50;evm.TU.scf7180000691956.1;evm.TU.scf7180000695611.13;evm.TU.scf7180000696023.116;evm.TU.scf7180000695652.51;evm.TU.scf7180000695652.16;evm.TU.scf7180000696055.381;evm.TU.scf7180000695652.53;evm.TU.scf7180000695611.5;evm.TU.scf7180000696059.111;evm.TU.scf7180000696017.18;evm.TU.scf7180000695611.8;evm.TU.scf7180000696023.23;evm.TU.scf7180000696017.43;evm.TU.scf7180000695652.48;evm.TU.scf7180000696023.41;evm.TU.scf7180000695681.168;evm.TU.scf7180000695652.21;evm.TU.scf7180000695611.32;evm.TU.scf7180000696023.61;evm.TU.scf7180000695903.47;evm.TU.scf7180000696023.3;evm.TU.scf7180000696131.10;evm.TU.scf7180000696015.10;evm.TU.scf7180000695611.25;evm.TU.scf7180000696131.73;evm.TU.scf7180000696023.36;evm.TU.scf7180000695540.24;evm.TU.scf7180000696055.285;evm.TU.scf7180000696020.35;evm.TU.scf7180000696035.21;evm.TU.scf7180000695760.19;evm.TU.scf7180000695970.199;evm.TU.scf7180000696	243

aae-miR-957:[54138_54207];	evm.TU.scf7180000696007.6;evm.TU.scf7180000696047.10;evm.TU.scf7180000696079.43;evm.TU.scf7180000688040.5;evm.TU.scf7180000690996.1;evm.TU.scf7180000695676.14;evm.TU.scf7180000696057.191;evm.TU.scf7180000694985.50;evm.TU.scf7180000695876.11;evm.TU.scf7180000695476.4;evm.TU.scf7180000696135.135;evm.TU.scf7180000696055.550;evm.TU.scf7180000696106.55;evm.TU.scf7180000695828.10;evm.TU.scf7180000695544.158;evm.TU.scf7180000695702.28;evm.TU.scf7180000695970.163;evm.TU.scf7180000695676.26;evm.TU.scf7180000695971.74;evm.TU.scf7180000688040.7;evm.TU.scf7180000690586.1;evm.TU.scf7180000695976.48;evm.TU.scf7180000696055.147;evm.TU.scf7180000695981.30;evm.TU.scf7180000695742.158;evm.TU.scf7180000696025.60;evm.TU.scf7180000695971.91;evm.TU.scf7180000695971.72;evm.TU.scf7180000696055.364;evm.TU.scf7180000695977.20;evm.TU.scf7180000696126.39;evm.TU.scf7180000696060.214;evm.TU.scf7180000695872.16;evm.TU.scf7180000691519.5;evm.TU.scf7180000695828.9;evm.TU.scf7180000690005.67;evm.TU.scf7180000696060.122;evm.TU.scf7180000695476.101;evm.TU.scf71800	45

aga-miR-263b:[97513_97603];aae-mir-263b:[97514_97593];	evm.TU.scf7180000696039.231;evm.TU.scf7180000695529.1;evm.TU.scf7180000696053.25;evm.TU.scf7180000694752.1;evm.TU.scf7180000696095.4;evm.TU.scf7180000696095.16;evm.TU.scf7180000696095.7;evm.TU.scf7180000696055.561;evm.TU.scf7180000689033.1;evm.TU.scf7180000696095.9;evm.TU.scf7180000695865.133;evm.TU.scf7180000694730.3;evm.TU.scf7180000696041.79;evm.TU.scf7180000695384.4;evm.TU.scf7180000696019.6;evm.TU.scf7180000689694.11;evm.TU.scf7180000696025.6;evm.TU.scf7180000696050.159;evm.TU.scf7180000696118.17;evm.TU.scf7180000696015.10;evm.TU.scf7180000695980.99;evm.TU.scf7180000689694.18;evm.TU.scf7180000696095.11;evm.TU.scf7180000695967.53;evm.TU.scf7180000695016.1;evm.TU.scf7180000695510.25;evm.TU.scf7180000695889.28;evm.TU.scf7180000696057.27;evm.TU.scf7180000689694.14;evm.TU.scf7180000696056.176;evm.TU.scf7180000695491.61;evm.TU.scf7180000694730.5;evm.TU.scf7180000695970.188;evm.TU.scf7180000690428.1;evm.TU.scf7180000696055.384;evm.TU.scf7180000696056.204;evm.TU.scf7180000695654.8;evm.TU.scf7180000695997.2;evm.TU.scf7180000695893.2	166

aga-miR-10:[711533_711634];cqu-mir-10:[711542_711621];api-mir-10:[711545_711621];isc-mir-10:[711546_711621];	evm.TU.scf7180000695920.55;evm.TU.scf7180000696010.9;evm.TU.scf7180000696078.9;evm.TU.scf7180000695705.191;evm.TU.scf7180000696060.3;evm.TU.scf7180000695038.10;evm.TU.scf7180000695681.137;evm.TU.scf7180000695519.9;evm.TU.scf7180000696109.4;evm.TU.scf7180000695750.24;evm.TU.scf7180000696118.3;evm.TU.scf7180000695890.2;evm.TU.scf7180000695750.11;evm.TU.scf7180000695750.34;evm.TU.scf7180000696034.39;evm.TU.scf7180000695556.101;evm.TU.scf7180000696046.25;evm.TU.scf7180000696135.116;evm.TU.scf7180000696057.119;evm.TU.scf7180000696076.14;evm.TU.scf7180000696039.99;evm.TU.scf7180000695978.3;evm.TU.scf7180000695750.16;evm.TU.scf7180000695750.4;evm.TU.scf7180000695636.2;evm.TU.scf7180000696104.9;evm.TU.scf7180000696016.16;evm.TU.scf7180000688816.1;evm.TU.scf7180000695750.1;evm.TU.scf7180000695750.2;evm.TU.scf7180000695047.4;evm.TU.scf7180000695568.16;evm.TU.scf7180000688412.1;evm.TU.scf7180000695750.6;evm.TU.scf7180000695473.46;evm.TU.scf7180000694995.4;evm.TU.scf7180000696058.13;evm.TU.scf7180000695709.98;evm.TU.scf7180000695680.99;evm.TU	58

aae-miR-2765:[27479_27563];	evm.TU.scf7180000695506.9;evm.TU.scf7180000695983.115;evm.TU.scf7180000695980.61;evm.TU.scf7180000696010.107;evm.TU.scf7180000695557.7;evm.TU.scf7180000696060.257;evm.TU.scf7180000695038.4;evm.TU.scf7180000695676.31;evm.TU.scf7180000694995.9;evm.TU.scf7180000695483.129;evm.TU.scf7180000696060.327;evm.TU.scf7180000696055.147;evm.TU.scf7180000696020.35;evm.TU.scf7180000696055.285;evm.TU.scf7180000695882.10;evm.TU.scf7180000695556.75;evm.TU.scf7180000695648.5;evm.TU.scf7180000695510.17;evm.TU.scf7180000695882.3;evm.TU.scf7180000696058.241;evm.TU.scf7180000696007.130;evm.TU.scf7180000695557.5;evm.TU.scf7180000696055.324;evm.TU.scf7180000696118.19;evm.TU.scf7180000695733.7;evm.TU.scf7180000696133.2;evm.TU.scf7180000695780.39;evm.TU.scf7180000695726.2;evm.TU.scf7180000696062.20;evm.TU.scf7180000696054.68;evm.TU.scf7180000696010.37;evm.TU.scf7180000695731.7;evm.TU.scf7180000696054.121;evm.TU.scf7180000695939.93;evm.TU.scf7180000696055.401;evm.TU.scf7180000686830.1;evm.TU.scf7180000694963.7;evm.TU.scf7180000691090.1;evm.TU.scf718000069611	141

cqu-miR-1175:[186451_186535];aae-mir-1175:[186455_186538];aga-mir-1175:[186457_186536];	evm.TU.scf7180000696065.12;evm.TU.scf7180000695742.36;evm.TU.scf7180000695695.9;evm.TU.scf7180000695536.3;evm.TU.scf7180000688499.2;evm.TU.scf7180000695869.5;evm.TU.scf7180000696131.121;evm.TU.scf7180000696015.118;evm.TU.scf7180000695934.12;evm.TU.scf7180000695536.4;evm.TU.scf7180000696135.23;evm.TU.scf7180000695787.71;evm.TU.scf7180000695536.2;evm.TU.scf7180000696050.187;evm.TU.scf7180000696013.40;evm.TU.scf7180000696017.18;evm.TU.scf7180000695971.4;evm.TU.scf7180000695934.13;evm.TU.scf7180000695844.8;evm.TU.scf7180000695593.1;evm.TU.scf7180000696015.106;evm.TU.scf7180000695970.159;evm.TU.scf7180000696131.144;evm.TU.scf7180000696012.35;evm.TU.scf7180000696039.3;evm.TU.scf7180000687150.1;evm.TU.scf7180000695939.165;evm.TU.scf7180000696056.37;evm.TU.scf7180000696050.193;evm.TU.scf7180000695799.22;evm.TU.scf7180000695983.58;evm.TU.scf7180000695544.69;evm.TU.scf7180000696060.431;evm.TU.scf7180000696073.10;evm.TU.scf7180000695787.87;evm.TU.scf7180000696059.98;evm.TU.scf7180000696048.79;evm.TU.scf7180000687033.1;evm.TU.scf7180000695544	104

aga-miR-929:[834_908];	evm.TU.scf7180000695983.156;evm.TU.scf7180000695870.1;evm.TU.scf7180000688324.1;evm.TU.scf7180000696027.110;evm.TU.scf7180000695947.30;evm.TU.scf7180000696059.134;evm.TU.scf7180000695411.1;evm.TU.scf7180000694985.12;evm.TU.scf7180000696133.53;evm.TU.scf7180000696127.11;evm.TU.scf7180000696060.85;evm.TU.scf7180000696131.169;evm.TU.scf7180000696055.515;evm.TU.scf7180000690675.4;evm.TU.scf7180000696029.53;evm.TU.scf7180000695557.121;evm.TU.scf7180000695920.56;evm.TU.scf7180000694214.1;evm.TU.scf7180000695681.225;evm.TU.scf7180000690675.10;evm.TU.scf7180000696049.104;evm.TU.scf7180000690675.1;evm.TU.scf7180000695483.129;evm.TU.scf7180000696045.114;evm.TU.scf7180000695979.1;evm.TU.scf7180000696042.8;evm.TU.scf7180000695799.22;evm.TU.scf7180000695893.29;evm.TU.scf7180000692168.1;evm.TU.scf7180000696126.97;evm.TU.scf7180000695661.21;evm.TU.scf7180000696010.106;evm.TU.scf7180000690675.3;evm.TU.scf7180000696106.48;evm.TU.scf7180000695690.149;evm.TU.scf7180000696060.345;evm.TU.scf7180000690675.9;evm.TU.scf7180000690675.2;evm.TU.scf71800006	123

aae-miR-276-1:[1458597_1458681];dya-mir-276a:[1458602_1458675];	evm.TU.scf7180000695920.55;evm.TU.scf7180000695939.173;evm.TU.scf7180000695561.9;evm.TU.scf7180000696056.3;evm.TU.scf7180000691397.6;evm.TU.scf7180000695708.33;evm.TU.scf7180000695549.73;evm.TU.scf7180000695549.74;evm.TU.scf7180000696040.83;evm.TU.scf7180000695506.27;evm.TU.scf7180000695549.19;evm.TU.scf7180000695681.189;evm.TU.scf7180000695538.8;evm.TU.scf7180000695549.76;evm.TU.scf7180000696052.94;evm.TU.scf7180000695947.40;evm.TU.scf7180000695549.22;evm.TU.scf7180000695549.96;evm.TU.scf7180000695702.40;evm.TU.scf7180000695903.30;evm.TU.scf7180000695501.5;evm.TU.scf7180000695053.1;evm.TU.scf7180000695702.37;evm.TU.scf7180000695971.115;evm.TU.scf7180000696048.144;evm.TU.scf7180000695549.62;evm.TU.scf7180000695939.41;evm.TU.scf7180000695702.35;evm.TU.scf7180000695549.11;evm.TU.scf7180000695705.209;evm.TU.scf7180000695545.8;evm.TU.scf7180000695506.25;evm.TU.scf7180000695702.44;evm.TU.scf7180000695549.16;evm.TU.scf7180000695702.38;evm.TU.scf7180000695691.2;evm.TU.scf7180000695702.46;evm.TU.scf7180000695549.90;evm.TU.scf718000069554	187

aga-miR-305:[15313_15399];cqu-mir-305:[15314_15398];	evm.TU.scf7180000695555.15;evm.TU.scf7180000695947.24;evm.TU.scf7180000695557.94;evm.TU.scf7180000695709.116;evm.TU.scf7180000696049.325;evm.TU.scf7180000695872.2;evm.TU.scf7180000695483.95;evm.TU.scf7180000690428.13;evm.TU.scf7180000696053.92;evm.TU.scf7180000689293.1;evm.TU.scf7180000695712.45;evm.TU.scf7180000696045.124;evm.TU.scf7180000689293.2;evm.TU.scf7180000695766.14;evm.TU.scf7180000695920.28;evm.TU.scf7180000695970.163;evm.TU.scf7180000695766.19;evm.TU.scf7180000696053.46;evm.TU.scf7180000695690.124;evm.TU.scf7180000695810.9;evm.TU.scf7180000689655.1;evm.TU.scf7180000689293.3;evm.TU.scf7180000695766.15;evm.TU.scf7180000695766.7;evm.TU.scf7180000696122.8;evm.TU.scf7180000696007.123;evm.TU.scf7180000696054.59;evm.TU.scf7180000695889.51;evm.TU.scf7180000696050.262;evm.TU.scf7180000696106.45;evm.TU.scf7180000695766.10;evm.TU.scf7180000696053.103;evm.TU.scf7180000695766.11;evm.TU.scf7180000695544.52;evm.TU.scf7180000696012.95;evm.TU.scf7180000696011.4;evm.TU.scf7180000695504.18;evm.TU.scf7180000696116.60;evm.TU.scf7180000696	73

aga-miR-927:[129424_129508];	evm.TU.scf7180000695865.171;evm.TU.scf7180000696050.40;evm.TU.scf7180000696112.15;evm.TU.scf7180000695851.3;evm.TU.scf7180000694106.4;evm.TU.scf7180000695694.10;evm.TU.scf7180000695742.123;evm.TU.scf7180000695476.79;evm.TU.scf7180000696000.13;evm.TU.scf7180000695980.65;evm.TU.scf7180000695705.22;evm.TU.scf7180000695851.18;evm.TU.scf7180000695980.79;evm.TU.scf7180000696000.6;evm.TU.scf7180000695851.26;evm.TU.scf7180000695980.76;evm.TU.scf7180000696022.42;evm.TU.scf7180000695980.58;evm.TU.scf7180000696050.183;evm.TU.scf7180000695627.6;evm.TU.scf7180000695705.49;evm.TU.scf7180000695971.74;evm.TU.scf7180000695980.43;evm.TU.scf7180000695971.115;evm.TU.scf7180000695476.134;evm.TU.scf7180000695556.144;evm.TU.scf7180000695476.53;evm.TU.scf7180000695980.88;evm.TU.scf7180000695476.125;evm.TU.scf7180000696051.103;evm.TU.scf7180000696051.42;evm.TU.scf7180000695476.130;evm.TU.scf7180000696023.32;evm.TU.scf7180000696013.19;evm.TU.scf7180000695920.17;evm.TU.scf7180000695476.1;evm.TU.scf7180000695476.54;evm.TU.scf7180000695980.112;evm.TU.scf718	293

cqu-miR-9:[660147_660251];aga-mir-9c:[660152_660241];aae-mir-9c:[660163_660237];	evm.TU.scf7180000695695.9;evm.TU.scf7180000696060.286;evm.TU.scf7180000695678.22;evm.TU.scf7180000695292.9;evm.TU.scf7180000695939.13;evm.TU.scf7180000695712.54;evm.TU.scf7180000695893.7;evm.TU.scf7180000696060.319;evm.TU.scf7180000695987.37;evm.TU.scf7180000695678.27;evm.TU.scf7180000696127.12;evm.TU.scf7180000696060.374;evm.TU.scf7180000695491.92;evm.TU.scf7180000696060.333;evm.TU.scf7180000695811.67;evm.TU.scf7180000696060.430;evm.TU.scf7180000696060.101;evm.TU.scf7180000695811.49;evm.TU.scf7180000696060.179;evm.TU.scf7180000695970.6;evm.TU.scf7180000696060.428;evm.TU.scf7180000696060.425;evm.TU.scf7180000696055.54;evm.TU.scf7180000696060.218;evm.TU.scf7180000695678.54;evm.TU.scf7180000695678.48;evm.TU.scf7180000696060.305;evm.TU.scf7180000696055.579;evm.TU.scf7180000696038.27;evm.TU.scf7180000696033.19;evm.TU.scf7180000696060.79;evm.TU.scf7180000695977.8;evm.TU.scf7180000696060.396;evm.TU.scf7180000689413.1;evm.TU.scf7180000695811.58;evm.TU.scf7180000695648.24;evm.TU.scf7180000696060.252;evm.TU.scf7180000695678.40;evm.TU.scf718	473

aga-miR-317:[7132_7206];	evm.TU.scf7180000696060.219;evm.TU.scf7180000689883.1;evm.TU.scf7180000696060.181;evm.TU.scf7180000695939.83;evm.TU.scf7180000695502.6;evm.TU.scf7180000695947.1;evm.TU.scf7180000695557.7;evm.TU.scf7180000696048.75;evm.TU.scf7180000695483.135;evm.TU.scf7180000696126.30;evm.TU.scf7180000695476.121;evm.TU.scf7180000696131.77;evm.TU.scf7180000695236.15;evm.TU.scf7180000693150.2;evm.TU.scf7180000695742.105;evm.TU.scf7180000688192.1;evm.TU.scf7180000695056.61;evm.TU.scf7180000695959.14;evm.TU.scf7180000688186.2;evm.TU.scf7180000696046.60;evm.TU.scf7180000695847.12;evm.TU.scf7180000696077.24;evm.TU.scf7180000696040.58;evm.TU.scf7180000696060.128;evm.TU.scf7180000693150.4;evm.TU.scf7180000695541.2;evm.TU.scf7180000696055.558;evm.TU.scf7180000695473.35;evm.TU.scf7180000695708.38;evm.TU.scf7180000695985.17;evm.TU.scf7180000695649.10;evm.TU.scf7180000695489.3;evm.TU.scf7180000696039.111;evm.TU.scf7180000695878.27;evm.TU.scf7180000696059.63;evm.TU.scf7180000693150.1;evm.TU.scf7180000695763.58;evm.TU.scf7180000696038.13;evm.TU.scf7180000696056.	58

cqu-miR-1891:[7473_7557];aae-mir-1891-2:[7479_7557];aga-mir-1891:[7479_7562];	evm.TU.scf7180000696060.417;evm.TU.scf7180000695489.15;evm.TU.scf7180000695947.36;evm.TU.scf7180000696060.90;evm.TU.scf7180000695562.17;evm.TU.scf7180000695742.184;evm.TU.scf7180000696038.83;evm.TU.scf7180000695988.9;evm.TU.scf7180000689884.1;evm.TU.scf7180000695666.1;evm.TU.scf7180000696070.41;evm.TU.scf7180000696050.99;evm.TU.scf7180000696054.162;evm.TU.scf7180000689180.37;evm.TU.scf7180000696118.3;evm.TU.scf7180000691091.1;evm.TU.scf7180000695939.42;evm.TU.scf7180000694926.2;evm.TU.scf7180000695939.8;evm.TU.scf7180000695504.53;evm.TU.scf7180000696079.78;evm.TU.scf7180000696054.130;evm.TU.scf7180000696062.29;evm.TU.scf7180000695763.67;evm.TU.scf7180000686919.1;evm.TU.scf7180000695732.18;evm.TU.scf7180000695483.25;evm.TU.scf7180000696008.12;evm.TU.scf7180000696036.11;evm.TU.scf7180000695944.7;evm.TU.scf7180000696057.51;evm.TU.scf7180000695537.5;evm.TU.scf7180000695970.24;evm.TU.scf7180000696024.65;evm.TU.scf7180000695811.10;evm.TU.scf7180000695483.56;evm.TU.scf7180000695977.29;evm.TU.scf7180000695502.7;evm.TU.scf7180000696130.79;	58

aga-miR-993:[748222_748341];	evm.TU.scf7180000696012.92;evm.TU.scf7180000696117.14;evm.TU.scf7180000695710.1;evm.TU.scf7180000695712.20;evm.TU.scf7180000695705.76;evm.TU.scf7180000696049.309;evm.TU.scf7180000696056.105;evm.TU.scf7180000696056.150;evm.TU.scf7180000695648.30;evm.TU.scf7180000695045.9;evm.TU.scf7180000695865.146;evm.TU.scf7180000695865.29;evm.TU.scf7180000696007.82;evm.TU.scf7180000696079.56;evm.TU.scf7180000696115.18;evm.TU.scf7180000696039.99;evm.TU.scf7180000695742.35;evm.TU.scf7180000696058.67;evm.TU.scf7180000696001.58;evm.TU.scf7180000690005.18;evm.TU.scf7180000695978.4;evm.TU.scf7180000696131.15;evm.TU.scf7180000695878.22;evm.TU.scf7180000696049.27;evm.TU.scf7180000695710.9;evm.TU.scf7180000695557.124;evm.TU.scf7180000696015.70;evm.TU.scf7180000695665.6;evm.TU.scf7180000696058.281;evm.TU.scf7180000695810.9;evm.TU.scf7180000696052.50;evm.TU.scf7180000696116.29;evm.TU.scf7180000696060.393;evm.TU.scf7180000696018.18;evm.TU.scf7180000695481.4;evm.TU.scf7180000694340.2;evm.TU.scf7180000695992.14;evm.TU.scf7180000696055.99;evm.TU.scf718000069602	53

aga-miR-13b:[1783317_1783392];	evm.TU.scf7180000695763.34;evm.TU.scf7180000696131.96;evm.TU.scf7180000695680.40;evm.TU.scf7180000695763.23;evm.TU.scf7180000695805.8;evm.TU.scf7180000695763.115;evm.TU.scf7180000696056.99;evm.TU.scf7180000695763.81;evm.TU.scf7180000695805.5;evm.TU.scf7180000695763.119;evm.TU.scf7180000695763.123;evm.TU.scf7180000695763.65;evm.TU.scf7180000695763.8;evm.TU.scf7180000695549.87;evm.TU.scf7180000696060.200;evm.TU.scf7180000695787.101;evm.TU.scf7180000695763.111;evm.TU.scf7180000695763.17;evm.TU.scf7180000695763.25;evm.TU.scf7180000696055.460;evm.TU.scf7180000695763.76;evm.TU.scf7180000695976.29;evm.TU.scf7180000695763.70;evm.TU.scf7180000695763.142;evm.TU.scf7180000695763.89;evm.TU.scf7180000696088.4;evm.TU.scf7180000695763.153;evm.TU.scf7180000696131.269;evm.TU.scf7180000695763.38;evm.TU.scf7180000696071.4;evm.TU.scf7180000695763.11;evm.TU.scf7180000695763.109;evm.TU.scf7180000695763.91;evm.TU.scf7180000695551.16;evm.TU.scf7180000690932.1;evm.TU.scf7180000695763.117;evm.TU.scf7180000695763.55;evm.TU.scf7180000695763.15;evm.TU.scf7180000	198

cqu-miR-184:[213388_213460];aga-mir-184:[213390_213471];dya-mir-184:[213400_213473];	evm.TU.scf7180000694908.5;evm.TU.scf7180000696005.8;evm.TU.scf7180000695871.13;evm.TU.scf7180000695934.49;evm.TU.scf7180000695681.137;evm.TU.scf7180000696127.12;evm.TU.scf7180000694837.6;evm.TU.scf7180000694837.5;evm.TU.scf7180000696056.15;evm.TU.scf7180000696025.93;evm.TU.scf7180000696127.15;evm.TU.scf7180000696127.6;evm.TU.scf7180000696127.14;evm.TU.scf7180000696127.20;evm.TU.scf7180000696135.8;evm.TU.scf7180000695713.31;evm.TU.scf7180000690005.68;evm.TU.scf7180000696055.22;evm.TU.scf7180000696127.5;evm.TU.scf7180000695526.17;evm.TU.scf7180000696013.123;evm.TU.scf7180000695556.39;evm.TU.scf7180000696058.35;evm.TU.scf7180000694837.4;evm.TU.scf7180000696045.124;evm.TU.scf7180000695569.31;evm.TU.scf7180000695678.25;evm.TU.scf7180000696089.28;evm.TU.scf7180000696127.19;evm.TU.scf7180000695538.17;evm.TU.scf7180000695369.2;evm.TU.scf7180000696005.107;evm.TU.scf7180000694914.1;evm.TU.scf7180000695287.10;evm.TU.scf7180000695556.155;evm.TU.scf7180000696049.259;evm.TU.scf7180000695941.7;evm.TU.scf7180000695562.29;evm.TU.scf7180000695980.	106

aga-miR-988:[126059_126142];	evm.TU.scf7180000696123.1;evm.TU.scf7180000694039.3;evm.TU.scf7180000695561.9;evm.TU.scf7180000696047.83;evm.TU.scf7180000696059.206;evm.TU.scf7180000695800.3;evm.TU.scf7180000696135.89;evm.TU.scf7180000696059.205;evm.TU.scf7180000695349.10;evm.TU.scf7180000696050.4;evm.TU.scf7180000686885.1;evm.TU.scf7180000695742.76;evm.TU.scf7180000696117.9;evm.TU.scf7180000695977.10;evm.TU.scf7180000696056.150;evm.TU.scf7180000695977.26;evm.TU.scf7180000696017.18;evm.TU.scf7180000687450.1;evm.TU.scf7180000696079.78;evm.TU.scf7180000696039.286;evm.TU.scf7180000696053.36;evm.TU.scf7180000695476.70;evm.TU.scf7180000695972.59;evm.TU.scf7180000687947.1;evm.TU.scf7180000696133.142;evm.TU.scf7180000696059.25;evm.TU.scf7180000696011.57;evm.TU.scf7180000695947.87;evm.TU.scf7180000695865.221;evm.TU.scf7180000696053.46;evm.TU.scf7180000696016.7;evm.TU.scf7180000696047.95;evm.TU.scf7180000694553.5;evm.TU.scf7180000696045.99;evm.TU.scf7180000695980.15;evm.TU.scf7180000696135.74;evm.TU.scf7180000696018.39;evm.TU.scf7180000696060.309;evm.TU.scf7180000690046.3	62

aae-miR-277:[25134_25224];aga-mir-277:[25135_25223];	evm.TU.scf7180000696010.9;evm.TU.scf7180000695537.15;evm.TU.scf7180000696054.164;evm.TU.scf7180000695681.362;evm.TU.scf7180000695987.44;evm.TU.scf7180000695510.15;evm.TU.scf7180000696028.51;evm.TU.scf7180000695661.1;evm.TU.scf7180000695712.26;evm.TU.scf7180000695537.13;evm.TU.scf7180000696059.46;evm.TU.scf7180000696057.192;evm.TU.scf7180000696043.85;evm.TU.scf7180000695712.92;evm.TU.scf7180000696126.2;evm.TU.scf7180000696055.387;evm.TU.scf7180000696046.28;evm.TU.scf7180000696005.119;evm.TU.scf7180000695653.109;evm.TU.scf7180000690005.18;evm.TU.scf7180000695947.39;evm.TU.scf7180000695537.12;evm.TU.scf7180000695537.2;evm.TU.scf7180000696037.3;evm.TU.scf7180000695274.3;evm.TU.scf7180000695977.34;evm.TU.scf7180000695537.17;evm.TU.scf7180000690488.1;evm.TU.scf7180000695537.16;evm.TU.scf7180000696059.201;evm.TU.scf7180000695056.37;evm.TU.scf7180000695510.5;evm.TU.scf7180000695562.29;evm.TU.scf7180000696051.16;evm.TU.scf7180000695537.10;evm.TU.scf7180000695510.23;evm.TU.scf7180000695510.9;evm.TU.scf7180000696115.11;evm.TU.scf718000069603	91

cqu-miR-315:[262642_262725];aga-mir-315:[262643_262718];	evm.TU.scf7180000696051.30;evm.TU.scf7180000696026.48;evm.TU.scf7180000696035.98;evm.TU.scf7180000695556.141;evm.TU.scf7180000696035.37;evm.TU.scf7180000696026.64;evm.TU.scf7180000695983.89;evm.TU.scf7180000696035.41;evm.TU.scf7180000695970.6;evm.TU.scf7180000696035.14;evm.TU.scf7180000696131.105;evm.TU.scf7180000696050.183;evm.TU.scf7180000695056.56;evm.TU.scf7180000696035.24;evm.TU.scf7180000696013.58;evm.TU.scf7180000695997.10;evm.TU.scf7180000695653.17;evm.TU.scf7180000695903.2;evm.TU.scf7180000695483.71;evm.TU.scf7180000696027.108;evm.TU.scf7180000696023.96;evm.TU.scf7180000696026.27;evm.TU.scf7180000696055.508;evm.TU.scf7180000696035.42;evm.TU.scf7180000696026.23;evm.TU.scf7180000695983.1;evm.TU.scf7180000695556.152;evm.TU.scf7180000696034.9;evm.TU.scf7180000695476.101;evm.TU.scf7180000696055.16;evm.TU.scf7180000695889.3;evm.TU.scf7180000696035.63;evm.TU.scf7180000696035.23;evm.TU.scf7180000696035.3;evm.TU.scf7180000695681.40;evm.TU.scf7180000696055.261;evm.TU.scf7180000695793.1;evm.TU.scf7180000696035.79;evm.TU.scf718000069	268

aga-miR-283:[23320_23411];	evm.TU.scf7180000696080.3;evm.TU.scf7180000693298.1;evm.TU.scf7180000695893.14;evm.TU.scf7180000696080.9;evm.TU.scf7180000695745.5;evm.TU.scf7180000695690.121;evm.TU.scf7180000690135.2;evm.TU.scf7180000695970.33;evm.TU.scf7180000696131.283;evm.TU.scf7180000690881.2;evm.TU.scf7180000696049.108;evm.TU.scf7180000696055.367;evm.TU.scf7180000696042.17;evm.TU.scf7180000695821.7;evm.TU.scf7180000696035.111;evm.TU.scf7180000690135.3;evm.TU.scf7180000696029.63;evm.TU.scf7180000695516.14;evm.TU.scf7180000695657.2;evm.TU.scf7180000696039.100;evm.TU.scf7180000695977.67;evm.TU.scf7180000696080.4;evm.TU.scf7180000695934.4;evm.TU.scf7180000695653.109;evm.TU.scf7180000695947.40;evm.TU.scf7180000695478.9;evm.TU.scf7180000696125.6;evm.TU.scf7180000696080.22;evm.TU.scf7180000695661.21;evm.TU.scf7180000696016.14;evm.TU.scf7180000696080.27;evm.TU.scf7180000690179.2;evm.TU.scf7180000696055.127;evm.TU.scf7180000691743.1;evm.TU.scf7180000695745.6;evm.TU.scf7180000695182.9;evm.TU.scf7180000696018.39;evm.TU.scf7180000695941.8;evm.TU.scf7180000696071.4;evm	191

aga-miR-7:[256539_256624];cqu-mir-7:[256548_256625];	evm.TU.scf7180000687555.9;evm.TU.scf7180000695581.5;evm.TU.scf7180000695902.2;evm.TU.scf7180000695932.46;evm.TU.scf7180000696048.61;evm.TU.scf7180000695987.15;evm.TU.scf7180000696319.1;evm.TU.scf7180000696060.419;evm.TU.scf7180000695742.47;evm.TU.scf7180000695980.60;evm.TU.scf7180000695536.2;evm.TU.scf7180000693473.3;evm.TU.scf7180000696049.283;evm.TU.scf7180000695702.40;evm.TU.scf7180000696056.142;evm.TU.scf7180000695702.37;evm.TU.scf7180000695541.72;evm.TU.scf7180000688731.1;evm.TU.scf7180000695537.1;evm.TU.scf7180000695702.35;evm.TU.scf7180000695702.44;evm.TU.scf7180000695702.38;evm.TU.scf7180000695536.5;evm.TU.scf7180000695702.46;evm.TU.scf7180000696032.59;evm.TU.scf7180000696026.19;evm.TU.scf7180000695680.8;evm.TU.scf7180000695702.2;evm.TU.scf7180000694399.21;evm.TU.scf7180000696056.204;evm.TU.scf7180000696051.89;evm.TU.scf7180000695681.20;evm.TU.scf7180000696047.131;evm.TU.scf7180000696044.17;evm.TU.scf7180000694922.20;evm.TU.scf7180000695970.198;evm.TU.scf7180000695801.20;evm.TU.scf7180000696060.339;evm.TU.scf7180000695865	137

aga-miR-79:[662987_663069];	evm.TU.scf7180000696079.3;evm.TU.scf7180000696065.12;evm.TU.scf7180000695528.2;evm.TU.scf7180000695876.2;evm.TU.scf7180000694302.4;evm.TU.scf7180000695684.12;evm.TU.scf7180000696045.150;evm.TU.scf7180000693308.1;evm.TU.scf7180000696045.70;evm.TU.scf7180000695893.7;evm.TU.scf7180000695742.168;evm.TU.scf7180000695720.13;evm.TU.scf7180000696130.71;evm.TU.scf7180000695720.23;evm.TU.scf7180000695900.9;evm.TU.scf7180000695686.1;evm.TU.scf7180000696043.66;evm.TU.scf7180000696126.78;evm.TU.scf7180000687597.5;evm.TU.scf7180000696050.89;evm.TU.scf7180000696077.24;evm.TU.scf7180000695971.62;evm.TU.scf7180000696055.446;evm.TU.scf7180000691900.2;evm.TU.scf7180000695992.2;evm.TU.scf7180000696079.6;evm.TU.scf7180000696055.51;evm.TU.scf7180000694302.2;evm.TU.scf7180000695747.19;evm.TU.scf7180000696058.127;evm.TU.scf7180000695720.1;evm.TU.scf7180000695550.1;evm.TU.scf7180000695953.10;evm.TU.scf7180000695876.12;evm.TU.scf7180000695787.40;evm.TU.scf7180000695876.7;evm.TU.scf7180000696055.571;evm.TU.scf7180000688698.1;evm.TU.scf7180000687555.7;evm.T	184

aga-miR-125:[1182085_1182192];cqu-mir-125:[1182096_1182185];	evm.TU.scf7180000695678.12;evm.TU.scf7180000695678.42;evm.TU.scf7180000695678.22;evm.TU.scf7180000695505.2;evm.TU.scf7180000695678.27;evm.TU.scf7180000695678.10;evm.TU.scf7180000695678.37;evm.TU.scf7180000696055.122;evm.TU.scf7180000695678.19;evm.TU.scf7180000695678.20;evm.TU.scf7180000695678.38;evm.TU.scf7180000695678.35;evm.TU.scf7180000695905.3;evm.TU.scf7180000695678.28;evm.TU.scf7180000695806.39;evm.TU.scf7180000696056.119;evm.TU.scf7180000695678.54;evm.TU.scf7180000695981.2;evm.TU.scf7180000695505.3;evm.TU.scf7180000696041.84;evm.TU.scf7180000695787.104;evm.TU.scf7180000695678.48;evm.TU.scf7180000688244.2;evm.TU.scf7180000696052.107;evm.TU.scf7180000688244.5;evm.TU.scf7180000695678.33;evm.TU.scf7180000696026.30;evm.TU.scf7180000695556.52;evm.TU.scf7180000695678.36;evm.TU.scf7180000695970.133;evm.TU.scf7180000695678.46;evm.TU.scf7180000696129.3;evm.TU.scf7180000695678.45;evm.TU.scf7180000695678.40;evm.TU.scf7180000695678.13;evm.TU.scf7180000695678.9;evm.TU.scf7180000688244.1;evm.TU.scf7180000695678.3;evm.TU.scf7180000695484	60

aga-miR-87:[1235293_1235384];	evm.TU.scf7180000696050.136;evm.TU.scf7180000695970.168;evm.TU.scf7180000695471.5;evm.TU.scf7180000695684.12;evm.TU.scf7180000696047.81;evm.TU.scf7180000695684.23;evm.TU.scf7180000695989.15;evm.TU.scf7180000696029.59;evm.TU.scf7180000695705.179;evm.TU.scf7180000696130.65;evm.TU.scf7180000696049.184;evm.TU.scf7180000696025.93;evm.TU.scf7180000695740.1;evm.TU.scf7180000695943.5;evm.TU.scf7180000696058.179;evm.TU.scf7180000695056.58;evm.TU.scf7180000695473.8;evm.TU.scf7180000695476.23;evm.TU.scf7180000694908.14;evm.TU.scf7180000695516.12;evm.TU.scf7180000696135.123;evm.TU.scf7180000695903.17;evm.TU.scf7180000696004.23;evm.TU.scf7180000695681.45;evm.TU.scf7180000695679.10;evm.TU.scf7180000696057.43;evm.TU.scf7180000696060.326;evm.TU.scf7180000696055.402;evm.TU.scf7180000695516.8;evm.TU.scf7180000696015.81;evm.TU.scf7180000695516.7;evm.TU.scf7180000695544.138;evm.TU.scf7180000695865.63;evm.TU.scf7180000696056.202;evm.TU.scf7180000695516.5;evm.TU.scf7180000696035.8;evm.TU.scf7180000695967.19;evm.TU.scf7180000695750.19;evm.TU.scf71800006	89

dgr-miR-124:[190102_190187];cqu-mir-124:[190106_190183];aae-mir-124:[190108_190183];aga-mir-124:[190108_190186];	evm.TU.scf7180000695705.297;evm.TU.scf7180000695705.257;evm.TU.scf7180000695705.99;evm.TU.scf7180000695519.18;evm.TU.scf7180000696063.1;evm.TU.scf7180000695473.41;evm.TU.scf7180000696050.239;evm.TU.scf7180000695705.288;evm.TU.scf7180000695705.115;evm.TU.scf7180000695705.326;evm.TU.scf7180000695705.1;evm.TU.scf7180000695705.13;evm.TU.scf7180000696125.6;evm.TU.scf7180000694833.11;evm.TU.scf7180000695705.204;evm.TU.scf7180000695483.115;evm.TU.scf7180000695705.231;evm.TU.scf7180000695705.245;evm.TU.scf7180000695705.331;evm.TU.scf7180000693723.4;evm.TU.scf7180000696055.508;evm.TU.scf7180000695708.12;evm.TU.scf7180000696032.59;evm.TU.scf7180000695705.253;evm.TU.scf7180000695705.315;evm.TU.scf7180000695705.77;evm.TU.scf7180000695705.303;evm.TU.scf7180000695920.21;evm.TU.scf7180000695980.105;evm.TU.scf7180000695705.218;evm.TU.scf7180000694990.1;evm.TU.scf7180000695705.280;evm.TU.scf7180000695544.135;evm.TU.scf7180000695705.153;evm.TU.scf7180000696055.417;evm.TU.scf7180000695980.55;evm.TU.scf7180000695705.309;evm.TU.scf7180000695705.237;e	323

aga-miR-1000:[961817_961909];	evm.TU.scf7180000696015.91;evm.TU.scf7180000695821.2;evm.TU.scf7180000695709.13;evm.TU.scf7180000695510.15;evm.TU.scf7180000695543.14;evm.TU.scf7180000696055.369;evm.TU.scf7180000695045.7;evm.TU.scf7180000696055.520;evm.TU.scf7180000695705.8;evm.TU.scf7180000695956.12;evm.TU.scf7180000696055.491;evm.TU.scf7180000695947.74;evm.TU.scf7180000696131.152;evm.TU.scf7180000694986.2;evm.TU.scf7180000696039.99;evm.TU.scf7180000696062.29;evm.TU.scf7180000695763.17;evm.TU.scf7180000696106.70;evm.TU.scf7180000695708.22;evm.TU.scf7180000695702.8;evm.TU.scf7180000696133.17;evm.TU.scf7180000695821.12;evm.TU.scf7180000695957.4;evm.TU.scf7180000695071.1;evm.TU.scf7180000695811.29;evm.TU.scf7180000696057.254;evm.TU.scf7180000696010.97;evm.TU.scf7180000695709.98;evm.TU.scf7180000695971.72;evm.TU.scf7180000695981.35;evm.TU.scf7180000695510.5;evm.TU.scf7180000696120.67;evm.TU.scf7180000695510.23;evm.TU.scf7180000695549.60;evm.TU.scf7180000695264.1;evm.TU.scf7180000695510.9;evm.TU.scf7180000696054.66;evm.TU.scf7180000695868.1;evm.TU.scf7180000696010.4	104

aga-miR-275:[27262_27344];	evm.TU.scf7180000696039.279;evm.TU.scf7180000693569.2;evm.TU.scf7180000688499.2;evm.TU.scf7180000695680.17;evm.TU.scf7180000695476.49;evm.TU.scf7180000688186.1;evm.TU.scf7180000696045.85;evm.TU.scf7180000692666.1;evm.TU.scf7180000687098.1;evm.TU.scf7180000695844.9;evm.TU.scf7180000695604.2;evm.TU.scf7180000691519.4;evm.TU.scf7180000695975.2;evm.TU.scf7180000695681.123;evm.TU.scf7180000695787.45;evm.TU.scf7180000695729.21;evm.TU.scf7180000695947.75;evm.TU.scf7180000696135.93;evm.TU.scf7180000695709.26;evm.TU.scf7180000695683.1;evm.TU.scf7180000695977.65;evm.TU.scf7180000695540.8;evm.TU.scf7180000695844.7;evm.TU.scf7180000696006.41;evm.TU.scf7180000696099.21;evm.TU.scf7180000695476.59;evm.TU.scf7180000696003.30;evm.TU.scf7180000695844.3;evm.TU.scf7180000696010.78;evm.TU.scf7180000696048.20;evm.TU.scf7180000687722.1;evm.TU.scf7180000695703.1;evm.TU.scf7180000687855.6;evm.TU.scf7180000695844.2;evm.TU.scf7180000695537.14;evm.TU.scf7180000695491.41;evm.TU.scf7180000687438.9;evm.TU.scf7180000695844.6;evm.TU.scf7180000696013.34;evm.TU.scf7	56

cqu-miR-iab-4:[295540_295614];aga-mir-iab-4:[295541_295620];aae-mir-iab-4-2:[295542_295614];	evm.TU.scf7180000690470.4;evm.TU.scf7180000695947.13;evm.TU.scf7180000695955.7;evm.TU.scf7180000696058.301;evm.TU.scf7180000696001.28;evm.TU.scf7180000695569.46;evm.TU.scf7180000695705.8;evm.TU.scf7180000696022.28;evm.TU.scf7180000695485.6;evm.TU.scf7180000695882.68;evm.TU.scf7180000696022.71;evm.TU.scf7180000695712.82;evm.TU.scf7180000696036.6;evm.TU.scf7180000695537.1;evm.TU.scf7180000695348.1;evm.TU.scf7180000695983.112;evm.TU.scf7180000694302.2;evm.TU.scf7180000696013.25;evm.TU.scf7180000696060.227;evm.TU.scf7180000696071.9;evm.TU.scf7180000696051.42;evm.TU.scf7180000695581.11;evm.TU.scf7180000696054.59;evm.TU.scf7180000696079.87;evm.TU.scf7180000696037.24;evm.TU.scf7180000688281.1;evm.TU.scf7180000696041.109;evm.TU.scf7180000696024.6;evm.TU.scf7180000696012.65;evm.TU.scf7180000695983.111;evm.TU.scf7180000695743.1;evm.TU.scf7180000695939.85;evm.TU.scf7180000696039.253;evm.TU.scf7180000695056.22;evm.TU.scf7180000696029.3;evm.TU.scf7180000695652.25;evm.TU.scf7180000696030.23;evm.TU.scf7180000696133.69;evm.TU.scf7180000696010.85	68

aga-let-7:[1181454_1181538];	evm.TU.scf7180000696045.102;evm.TU.scf7180000696045.93;evm.TU.scf7180000695934.49;evm.TU.scf7180000696051.22;evm.TU.scf7180000696027.65;evm.TU.scf7180000696027.117;evm.TU.scf7180000696045.152;evm.TU.scf7180000696045.139;evm.TU.scf7180000696030.46;evm.TU.scf7180000696045.91;evm.TU.scf7180000696045.72;evm.TU.scf7180000695989.15;evm.TU.scf7180000696027.69;evm.TU.scf7180000696027.59;evm.TU.scf7180000696027.2;evm.TU.scf7180000696045.36;evm.TU.scf7180000696027.122;evm.TU.scf7180000695710.3;evm.TU.scf7180000695939.3;evm.TU.scf7180000696027.61;evm.TU.scf7180000695740.1;evm.TU.scf7180000696027.72;evm.TU.scf7180000696045.44;evm.TU.scf7180000696131.152;evm.TU.scf7180000696118.23;evm.TU.scf7180000696045.56;evm.TU.scf7180000696045.82;evm.TU.scf7180000696027.4;evm.TU.scf7180000696045.135;evm.TU.scf7180000696053.46;evm.TU.scf7180000696027.115;evm.TU.scf7180000696045.77;evm.TU.scf7180000696045.21;evm.TU.scf7180000696027.51;evm.TU.scf7180000696045.41;evm.TU.scf7180000696045.133;evm.TU.scf7180000696045.60;evm.TU.scf7180000696045.14;evm.TU.scf718000	214

aae-miR-9b:[663587_663666];	evm.TU.scf7180000696035.98;evm.TU.scf7180000696048.120;evm.TU.scf7180000696054.120;evm.TU.scf7180000695893.14;evm.TU.scf7180000695684.12;evm.TU.scf7180000696035.37;evm.TU.scf7180000696063.1;evm.TU.scf7180000696054.93;evm.TU.scf7180000696054.159;evm.TU.scf7180000695686.1;evm.TU.scf7180000696035.41;evm.TU.scf7180000696054.31;evm.TU.scf7180000696130.2;evm.TU.scf7180000695859.2;evm.TU.scf7180000696035.14;evm.TU.scf7180000693169.2;evm.TU.scf7180000695681.212;evm.TU.scf7180000693169.7;evm.TU.scf7180000696035.24;evm.TU.scf7180000696054.91;evm.TU.scf7180000696054.118;evm.TU.scf7180000695872.30;evm.TU.scf7180000695872.8;evm.TU.scf7180000696054.196;evm.TU.scf7180000696131.10;evm.TU.scf7180000695648.24;evm.TU.scf7180000696054.96;evm.TU.scf7180000696055.431;evm.TU.scf7180000696054.41;evm.TU.scf7180000696054.32;evm.TU.scf7180000695872.18;evm.TU.scf7180000696035.42;evm.TU.scf7180000696054.5;evm.TU.scf7180000696055.402;evm.TU.scf7180000686966.1;evm.TU.scf7180000692471.6;evm.TU.scf7180000696054.142;evm.TU.scf7180000695652.10;evm.TU.scf718000068	412

cqu-miR-281:[2646127_2646221];aga-mir-281:[2646128_2646223];aae-mir-281:[2646130_2646221];	evm.TU.scf7180000696131.96;evm.TU.scf7180000694302.4;evm.TU.scf7180000696060.148;evm.TU.scf7180000693124.2;evm.TU.scf7180000695787.12;evm.TU.scf7180000695023.1;evm.TU.scf7180000696115.2;evm.TU.scf7180000696120.41;evm.TU.scf7180000695341.1;evm.TU.scf7180000695780.58;evm.TU.scf7180000696115.19;evm.TU.scf7180000690005.21;evm.TU.scf7180000695865.115;evm.TU.scf7180000696050.112;evm.TU.scf7180000695872.22;evm.TU.scf7180000695882.7;evm.TU.scf7180000696055.561;evm.TU.scf7180000696025.25;evm.TU.scf7180000695705.35;evm.TU.scf7180000696057.192;evm.TU.scf7180000691207.1;evm.TU.scf7180000695865.52;evm.TU.scf7180000695981.7;evm.TU.scf7180000695980.60;evm.TU.scf7180000695710.3;evm.TU.scf7180000695747.27;evm.TU.scf7180000695236.23;evm.TU.scf7180000695981.1;evm.TU.scf7180000696130.2;evm.TU.scf7180000695981.18;evm.TU.scf7180000696115.3;evm.TU.scf7180000695981.40;evm.TU.scf7180000696005.36;evm.TU.scf7180000694695.1;evm.TU.scf7180000692432.2;evm.TU.scf7180000695903.68;evm.TU.scf7180000696115.26;evm.TU.scf7180000695872.30;evm.TU.scf7180000695872.8;ev	242

aga-miR-278:[4349218_4349300];	evm.TU.scf7180000696028.45;evm.TU.scf7180000696028.10;evm.TU.scf7180000695706.1;evm.TU.scf7180000695608.15;evm.TU.scf7180000695473.32;evm.TU.scf7180000696028.61;evm.TU.scf7180000696027.135;evm.TU.scf7180000696041.17;evm.TU.scf7180000694985.12;evm.TU.scf7180000696107.5;evm.TU.scf7180000696047.19;evm.TU.scf7180000696028.68;evm.TU.scf7180000695742.47;evm.TU.scf7180000696126.43;evm.TU.scf7180000695937.3;evm.TU.scf7180000696035.56;evm.TU.scf7180000695536.2;evm.TU.scf7180000695475.3;evm.TU.scf7180000695980.63;evm.TU.scf7180000687150.1;evm.TU.scf7180000696051.139;evm.TU.scf7180000696032.13;evm.TU.scf7180000695556.102;evm.TU.scf7180000696013.84;evm.TU.scf7180000695476.32;evm.TU.scf7180000696030.11;evm.TU.scf7180000696028.5;evm.TU.scf7180000696028.30;evm.TU.scf7180000695536.5;evm.TU.scf7180000695510.23;evm.TU.scf7180000696059.142;evm.TU.scf7180000696025.69;evm.TU.scf7180000696028.64;evm.TU.scf7180000695818.2;evm.TU.scf7180000696028.66;evm.TU.scf7180000696055.125;evm.TU.scf7180000695510.6;evm.TU.scf7180000696028.9;evm.TU.scf7180000695680.143	146
		6080
